# Epimerisation in Peptide Synthesis

**DOI:** 10.3390/molecules28248017

**Published:** 2023-12-08

**Authors:** Suleman Duengo, Muhamad Imam Muhajir, Ace Tatang Hidayat, Weny J. A. Musa, Rani Maharani

**Affiliations:** 1 Department of Chemistry, Faculty of Mathematics and Natural Sciences, Universitas Padjadjaran, Sumedang 45363, West Java, Indonesia; sulemanduengo@ung.ac.id (S.D.); imamisaa123@gmail.com (M.I.M.); ace.hidayat@unpad.ac.id (A.T.H.); 2 Department of Chemistry, Faculty of Mathematics and Natural Sciences, Universitas Negeri Gorontalo, Gorontalo 96128, North Sulawesi, Indonesia; weny@ung.ac.id; 3 Central Laboratory, Universitas Padjadjaran, Sumedang 45363, West Java, Indonesia; 4 Research Collaboration Centre for Theranostic Radiopharmaceutical, National Research and Innovation Agency (BRIN), Sumedang 45363, West Java, Indonesia

**Keywords:** epimerisation, side reaction, peptide synthesis, cyclisation, solid-phase peptide synthesis

## Abstract

Epimerisation is basically a chemical conversion that includes the transformation of an epimer into another epimer or its chiral partner. Epimerisation of amino acid is a side reaction that sometimes happens during peptide synthesis. It became the most avoided reaction because the process affects the overall conformation of the molecule, eventually even altering the bioactivity of the peptide. Epimerised products have a high similarity of physical characteristics, thus making it difficult for them to be purified. In regards to amino acids, epimerisation is very important in keeping the chirality of the assembled amino acids unchanged during the peptide synthesis and obtaining the desirable product without any problematic purification. In this review, we report several factors that induce epimerisation during peptide synthesis, including how to characterise and affect the bioactivities. To avoid undesirable epimerisation, we also describe several methods of suppressing the process.

## 1. Introduction

**Figure 1:**
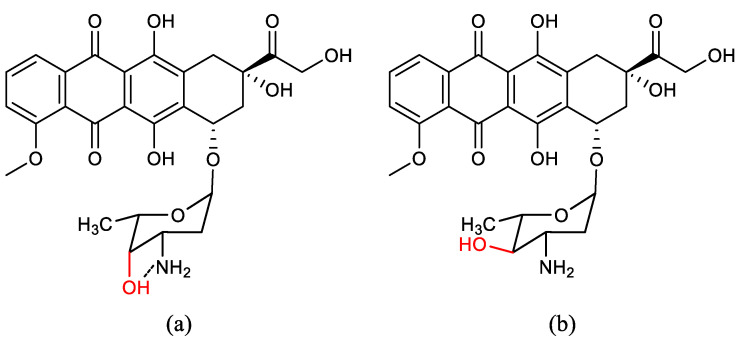
Structure of (**a**) doxorubicin and (**b**) epirubicin (Paul Launchbury and Habboubi, 1993).

Epimerisation is a process of epimer formation in which stereoisomers have different configurations at only one of the stereogenic centres [1]. Epimerisation is basically a chemical conversion that includes the transformation of an epimer into another epimer or its chiral partner [2]. Doxorubicin and epirubicin are one pair of epimers (Figure 1). The fundamental difference between epirubicin and doxorubicin can be observed in the epimerisation of the 4′-C atoms, in which the 4′-OH group of doxorubicin is attached to the carbon with an axial configuration, while the 4′-OH group of epirubicin is attached to the carbon with the equatorial configuration. This difference, where there is a small change at only one carbon configuration without any changes in the molecular weight and molecular formula, totally affects their biological properties [2].

Other findings also revealed that the epimer pairs can show different bioactivities. [3] reported that the presence of D-amino acid in the structure increases structural or conformational flexibility compared to the L-isomer. Incorporation of D-amino acid can enhance biological activity by stabilising certain molecular conformations [3]. For example, cyclopentapeptide, an astin-C analogue containing a D-amino acid residue, exhibits better immunosuppressant activity than its isomer. Li et al. (2018) also said that, in the case of omega-agatoxin IVB and IVC, the peptide with D-serine at position 46 was four times more potent than the L-serine isomer in its inhibitory action on P-type Ca channels in Purkinje cells [4]. These are several findings showing how the epimerisation process could be an important issue in medicinal chemistry. 

**Figure 2:**
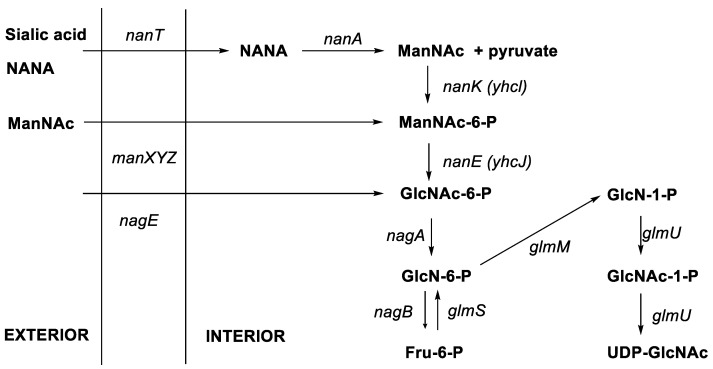
Epimerisation of N-acetylglucosamine (GlcNAc) into N-acetylmannosamine (NANA) catalysed by a renin-binding protein [5].

**Figure 3:**
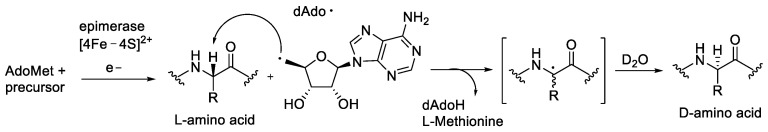
Proposed epimerisation of amino acid by AdoMet radical proteusin epimerases [6].

Generally, epimerisation is a slow and spontaneous reaction. Mostly, the process can be catalysed by enzymes [5]. Post-translational epimerisation of selective amino acids in peptide chains is a difficult challenge, requiring suitable enzymes [6]. For example, the conversion of N-acetylglucosamine to N-acetylmannosamine is an epimerisation reaction that occurs in the presence of a renin-binding protein that acts as a catalyst (Figure 2) [7]. Further, the radical enzyme of S-adenosyl-*L-*methionine (AdoMet) epimerase is able to catalyse the conversion of *L-*amino acids to *D-*amino acids in the synthesis of ribosomal peptides (Figure 3) [8].

In peptide synthesis, amino acid epimerisation is one of the most avoided reactions, as it is known that the biological activity of peptides is very dependent on their conformation with dimensional atomic alignment [9]. Additionally, epimerisation of one amino acid in the peptide sequence could affect the overall conformation of the molecule, eventually affecting the bioactivity of the peptide. Epimerised products have a high similarity of physical characteristics [10]. The epimerisation product further increases the difficulty of purification [11].

**Figure 4:**
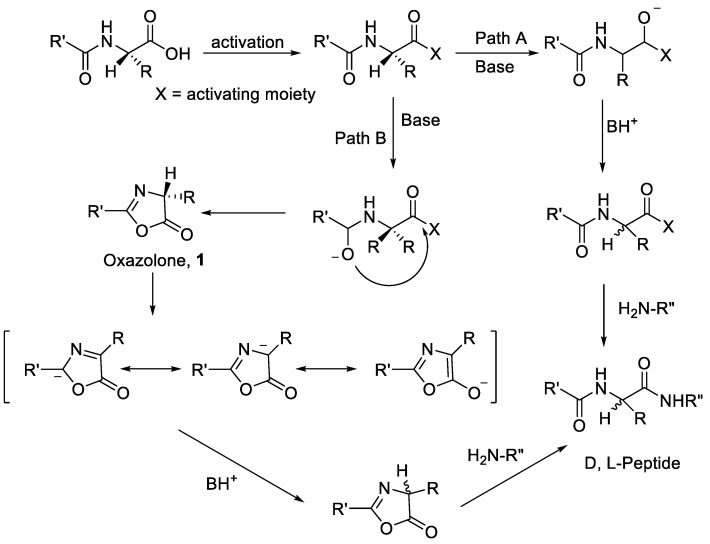
Mechanism of epimerisation/racemisation through oxazolone intermediate [13].

Generally, there are two general mechanisms of epimerisation in peptide synthesis. The first mechanism is observed through the formation of oxazol-5(4H)-one, which occurs through a strong activation of the carboxylate group backbone on the protected amino acid and might induce the formation of oxazol-5(4H)-one intermediate. The Nα attack from the same amino acid or peptide could lead to the epimerisation product (Figure 4). The path B mechanism was regarded as the most predominant source of amino acid racemisation during peptide synthesis [12].

The second mechanism is epimerisation via direct Hα abstraction by bases in amino acid. This epimerisation process is possible in peptide synthesis, especially with amino acid residues with high acidic Hα. The alpha proton of amino acid is abstracted by a base, producing anionic amino acid during the activation or deprotection process in a base-mediated condition (Figure 4 path A). The re-protonation of the generated anionic amino acid by proton sources leads to the amino acid racemisation [14].

Based on the introduction above, epimerisation in peptide synthesis can be an issue in drug discovery as it can lead to products that have totally different biological activities. In peptide synthesis, epimerisation has become a significant synthetic problem that needs to be recognised and overcome by peptide chemists. In this review, we describe several factors that induce epimerisation during peptide synthesis, in addition to several strategies designed to suppress epimerisation.

## 2. Epimerisation-Inducing Factors

### 2.1. Activation of Carboxylic Groups to Active Esters

In the structure of a peptide, amino acids are linked via amide bonds between the carboxyl group of one amino acid and the α-amino group of another. Emil Fischer, who first proposed this structure, called this amide bond a peptide bond [15]. The peptide bond formed between the two amino acids can usually be observed in a condensation reaction between the carboxyl group of an amino acid and the amino group of another amino acid by releasing a water molecule. However, a carboxylic group on an amino acid is less reactive, so in the process of peptide bond formation, an activating reagent is mostly required. In this case, a coupling reagent is used, which functions to activate the carboxylic group on the amino acid to become an active ester so that it can be attacked by other amino groups [16]. In the mechanism, the carboxylic group on the peptide is activated using a coupling reagent and forms an activated product. Unfortunately, this activation step, along with the next coupling reaction, has an issue, which is the potential loss of chiral integrity upon the carboxyl residue undergoing activation [13,17]. Several activated intermediates react directly with other amino acids to form a peptide bond, while several intermediates form an oxazol-5(4H)-one derivative, leading to epimerised or racemised products. This process allows the formation of two products, which are a target peptide together with the epimerised or racemised products [18].

As described in the introduction, there are two major pathways for the loss of configuration where both pathways are basic catalysed, which are (a) direct enolisation (path A) and (b) 5-(4H)-oxazolone formation (path B). Both pathways could induce the epimerisation or racemisation, as described in Figure 4 [19].

**Figure 5:**
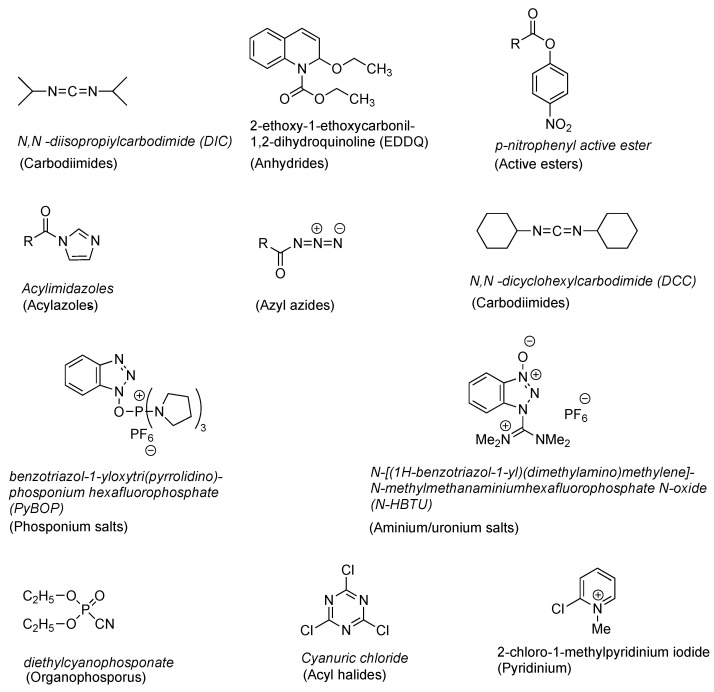
Various types of coupling reagents.

Various types of coupling reagents in the activation process reportedly could lead to the racemisation and epimerisation process. Several types of coupling reagents have been discovered in the solid-phase synthesis of peptides, such as carbodiimides, anhydrides, active esters, acylazoles, acyl azides, acyl halides, phosphonium salts, uronium salts, organophosphorus, triazine, and pyridinium (Figure 5) [17]. 

**Figure 6:**
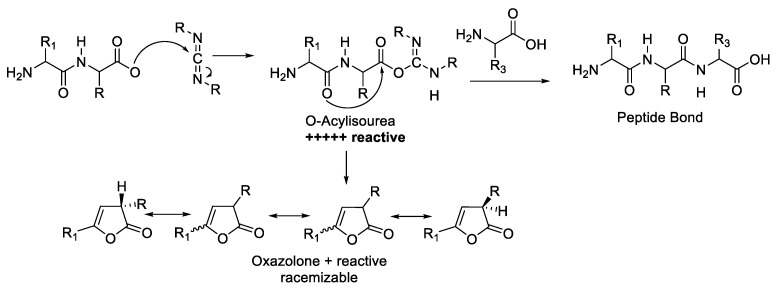
Reactive O-Acylisourea ester increases racemisation.

One carbodiimide-based coupling reagent is DCC (*N*,*N*′-dicyclohexylcarbodiimide) (Figure 5). Activation of the carboxylic group using DCC produces ortho-acylurea, which is very reactive. The reactive intermediate could potentially form an oxazolone, potentially triggering the racemisation (Figure 6) [20].

**Figure 7:**
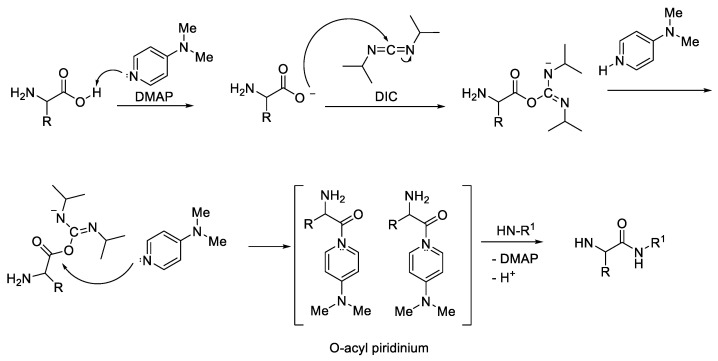
DIC/DMAP coupling agent mechanism.

DMAP (4-dimethylaminopyridine) is mostly added to the reaction to form acyl pyridinium (Figure 7), which can suppress the racemisation product [19].

**Figure 8:**
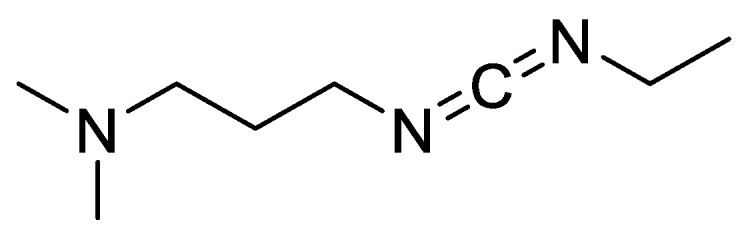
Structure of 1-ethyl-3-[3-(dimethylaminopropy1) carbodiimide] (EDC).

**Figure 9:**
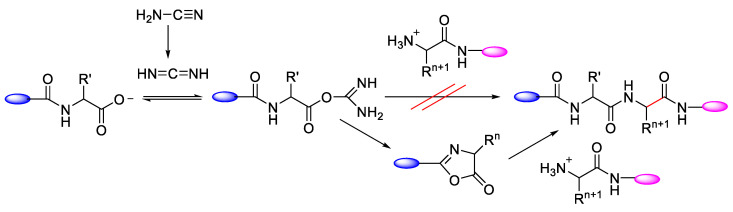
Epimerisation mechanism using carbodiimide coupling agent.

Dunetz (2016) reported that EDC [1-ethyl-3-[3-(dimethylaminopropy1) carbodiimide] (Figure 8) tends to cause epimerisation more than DCC (Figure 9) [21]. A good performance of both EDC and DCC was shown when these carbodiimides were mixed with HOBt, which has been proven to suppress epimerisation (Table 1).

The results in Table 1 indicate that, in terms of preservation of stereochemistry, there is at most a marginal difference between EDC and DCC in the induction of epimerisation. EDC was found to more likely lead to racemisation (25%) than DCC.

Another study also reports that the EDC coupling reagent induced more epimerisation than the DIC coupling reagent (Table 2) [22]. Comparisons between EDC, EDC-HCI, and DIC during condensation often showed cleaner reactions in the latter two cases [23]. The results may be related to the presence of an unhindered basic amino function in EDC structures. A negative effect of a base on loss of configuration would be particularly important for slow coupling processes. Thus, in the solid-phase peptide synthesis of GIy-Phe-Pro-NH_2_, the following epimerisation percentages were observed: EDC/HOAt (29.8%); EDC-HC1/HOAt (24.1%); DIC/HOAt (4.2%) (Tabel 2) [22].

**Figure 10:**
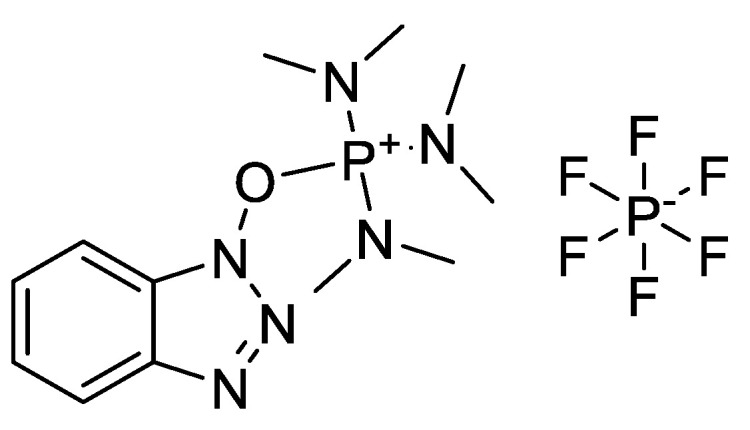
BOP coupling reagent.

Spatola et al. synthesised cyclo(D-Trp-D-Asp-Pro-D-Val-Leu), also known as BQ-123, a recently characterised cyclic pentapeptide endothelin antagonist, in 1996 (Table 3). This target used a limited mixture of cyclic pentapeptides. The racemisation was examined throughout the synthesis of the four analogues of cyclo[Xxx-D-Leu-Val-D-Pro-Asp], where Xxx = Ala, Phe, Tyr, and Trp [24]. To do this, the stereochemistry of hydrolysed amino acids was routinely examined using Marfey’s reagent, Na-(2,4-dinitro-S-fluorophenyl)-L-alaninamide. Racemisation was hypothesised to most likely occur during the transition of the linear precursor into the cyclic product and during the activation of the linear peptide’s C-terminus utilising a BOP active ester (Figure 10) [25]. 

### 2.2. Amino Acids

Racemisation can also be induced by the structure of the amino acids. It occurs due to the presence of a stereochemical centre in the amino acid structure [26].

**Figure 11:**
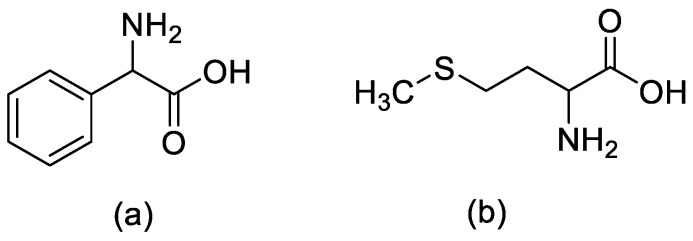
Structures of (**a**) phenylglycine and (**b**) methionine.

Among the amino acids that have the potential to cause racemisation are phenylglycine [27] and methionine [3] (Figure 11). The anion produced upon H-alpha abstraction on phenylglycine amino acid can be stabilised by the aryl side chain group and increases the possibility of epimerisation. Thus, all the amino acids with electron-withdrawing group side chains can undergo epimerisation [28].

**Figure 12:**
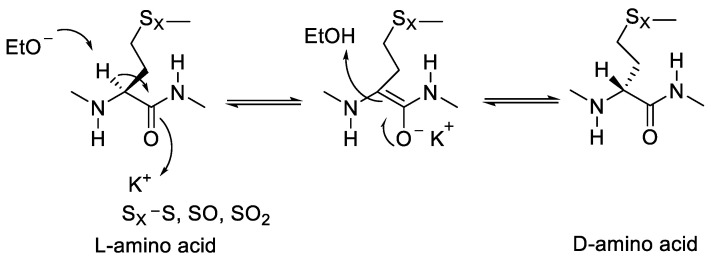
Proton abstraction of methionine by base.

Cyclisation of peptides featuring several sequential N-methyl-methionines was reported to involve a risk of epimerisation. The proton alpha of methionine residue makes it easy to direct abstraction under basic conditions during the activation step. This condition can lead to a direct epimerisation mechanism, as shown in Figure 12. Further, Jadhav et al. (2021) found that amino acid residues such as Leu, Ile, Phe, and Val have similar acidity to that of Met, so the methionine epimerisation (Figure 12) was not considered to be kinetically more preferable than the other amino acid residues in cyclic peptides. Epimerisation being caused by other residues such as Leu, Ile, Phe, and Val is also possible.

**Figure 13:**
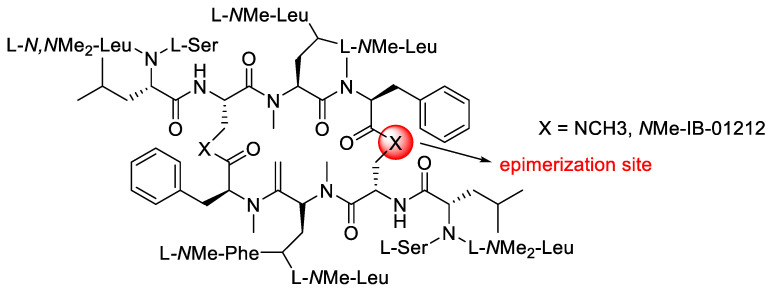
Structure of NMe-IB-01212.

The linear octapeptide [Boc-L-Dap(Me&1)-NMe-Leu-NMe-Phe&2] (Figure 13) was treated under various cyclisation conditions to find a cyclisation condition with minimal C-epimerisation (Table 4). Cyclisation failed to occur at neutral conditions (entry 4), and gave the result when it was carried out at pH 8. The octapeptide precursor was consequently dissolved in DMF and cyclised with PyBOP and HOAt while maintaining pH 8 with the addition of DIEA. Despite the rigidity imposed by a number of N-methyl amino acids, the cyclisation was entirely successful in 3 h. The best result was obtained using DIEA as the base and DMF as the solvent, where the cyclisation resulted in the highest yield and the lowest percentage of epimerisation at the C-terminus (entry 3) [29].

**Figure 14:**
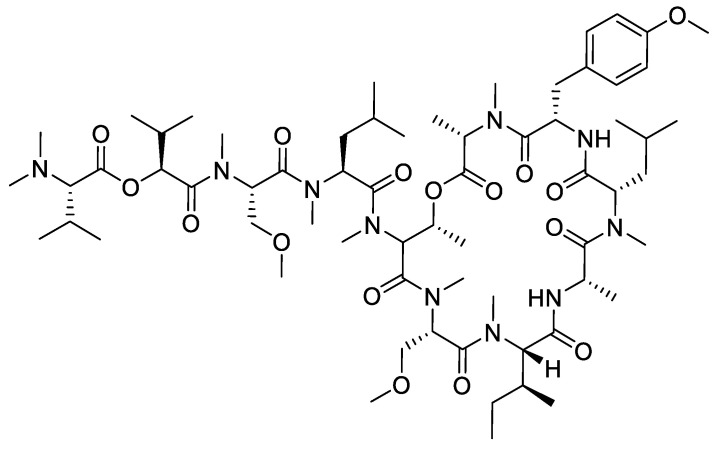
Structure of coibamide A.

A different case was reported by Nabika et al. (2015) in the synthesis of coibamide A (Figure 14), which has eight *N*-methylamino acids and two ester bonds. Coibamide A (1) is a cyclic depsipeptide that was originally isolated from a Panamanian marine cyanobacterium. This compound has been reported to exhibit potent antiproliferative activity against a number of human cell lines, where it operates according to a unique mechanism of action [30].

Synthesis of coibamide A was carried out in the solid phase using a series of different coupling reagents, including the use of DIC/HOAt, HATU, and BTC systems to optimise the coupling conditions, particularly for any coupling involving the N-methylamino acid.

Using a HATU/DIPEA system, Tyr(Me)1 and Ala residues were successfully linked to N-methylamino acids. By comparison, MeLeu and MeIle were successfully linked onto unmethylated amino acids when the typical Fmoc-based Solid Phase Peptide Synthesis (SPPS) procedure (DIC/HOBt) was applied. The partial epimerisation of the product (diastereomer excess = 72:28) was found during the coupling of MeSer(Me) onto the N-methylamino group of MeIle7 in the presence of HATU/DIPEA. This finding was attributed to the slow reaction of the two coupling partners under the basic conditions. By using DIC/HOAt in a non-basic condition, epimerisation was not observed even though the coupling reaction was not completed. On the other hand, coupling of Fmoc-Ser(Me)-OH was successfully completed using the same DIC/HOAt reagents.

Liang et al. (2017) observed a racemisation of phenylglycine in Fmoc-based solid-phase peptide synthesis [31]. Phenylglycine-containing peptide has been known to have potential pharmaceutical applications and, in regards to chemical biology, there are important building blocks within various antimicrobial peptides, such as streptogramins (virginiamycin S, streptogramin B, pristinamycin I, or dityromycin) or glycopeptides (vancomycin) [32]. Besides having a potential biological activation, obtaining the correct epimer of Phg-containing peptide is challenging because of the increased acidity of the proton at the α-carbon, which facilitates the loss of configuration during synthesis.

**Figure 15:**
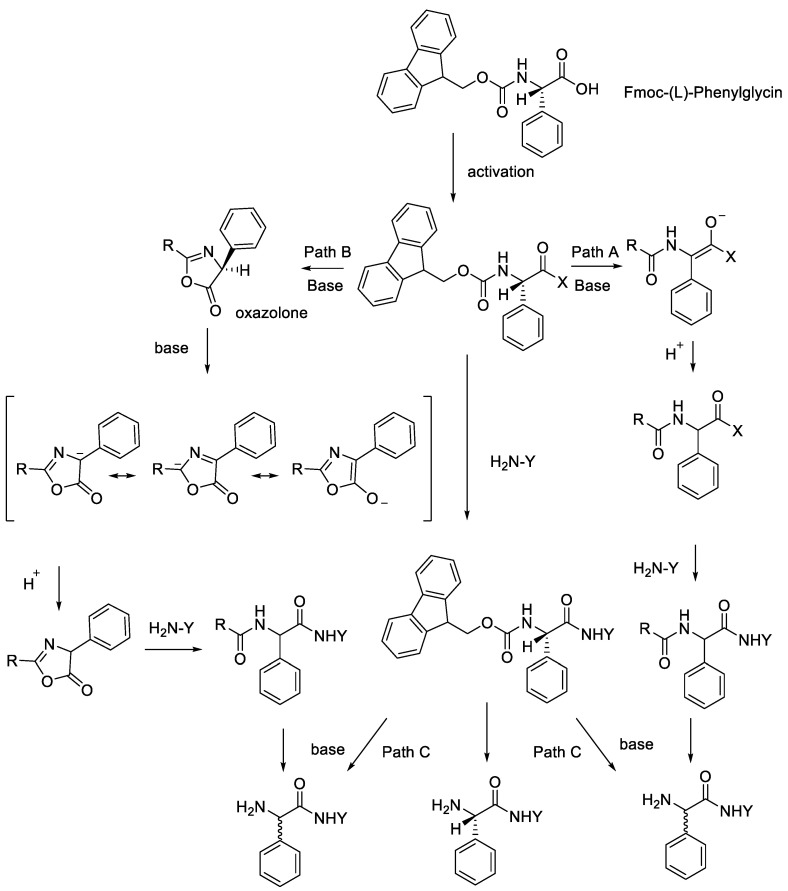
Potential racemisation pathways of Fmoc-(*L*)-phenylglycine during solid-phase peptide synthesis.

Figure 15 illustrates this proposal. Due to the acidity of the a-carbon, it is suggested that the racemisation of Phg during SPPS proceed via enolisation under basic circumstances that are encountered during coupling (pathways A and B) or Fmoc deprotection (pathway C) phases. The kind and position of the phenyl ring substituents determine the intensity of the mesomeric action and the consequent stability of the anion intermediate at the −carbon, which likely explains the sequence of sensitivity to racemisation in Phg amino acid.

**Figure 16:**
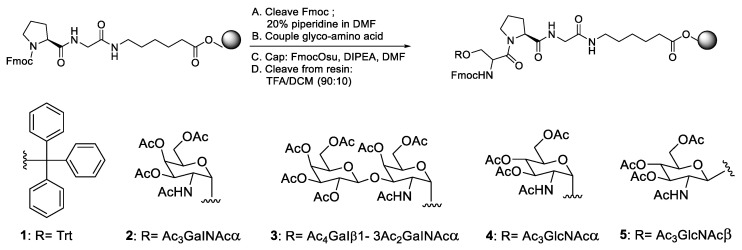
Epimerisation study in glycopeptide synthesis.

Other research involves the synthesis of glycopeptides (Figure 16), which are used extensively in basic research [33], and an investigation of various clinical applications, such as antimicrobe [34], anticancer [35], vaccine [36], and antigen [37] uses. Zhang et al. (2012) synthesised and optimised the four Fmoc-Ser amino acids that are commonly found in O-linked glycans [38]. Fmoc-Ser-Trt was also optimised because it had previously been found to have a higher rate of racemisation than most other natural amino acids [39]. The attachment Fmoc-Ser-Glycan was carried out using several coupling agents and was evaluated in a racemisation formation.

From Table 5, it can be seen that the condition of 3.3 equivalent of amino acid and HATU/HOAT 3.65/3.70 equivalent with the presence of 7.2 equivalent basic DIPEA in DMF showed a significant difference in epimerisation between several Fmoc-Ser(R)-OH amino acid examples, whereas Fmoc-Ser(Ac_3_GaINAca) (0.8%) and Fmoc-Ser(Ac_3_GlcNAcb)-OH (2.0%) showed a minor value epimerisation yield. Fmoc-Ser(Ac_4_GaIb1-3Ac_2_GaINAca)-OH (65.6%) and Fmoc-Ser(Ac_3_GlcNAca)-OH (72.5%) showed more epimerisation products than the other side chain in the same coupling condition. On the other hand, Fmoc-Ser(Trt)-OH also showed a high epimerisation yield in the condition of 4.4 equivalent of amino acid and HATU/HOAT 4.0 equivalent with the presence of 8.8 equivalent basic NMM in DMF solvent. From these results, it can be concluded that glycol-amino acids show increased epimerisation, especially in Fmoc-Ser(R)-OH amino acid. The best coupling condition can suppress the epimerisation yield.

Another study reported that synthesis of 5,6-dihydro-4H-1,3-thiazine containing peptide mimics also results in epimerisation during amide bond formation [40]. The 5-membered heterocycles, 1,3-thiazolines and 1,3-thiazoles, are the most prevalently studied thioimidate isosteres for the peptide bond [41].

**Figure 17:**
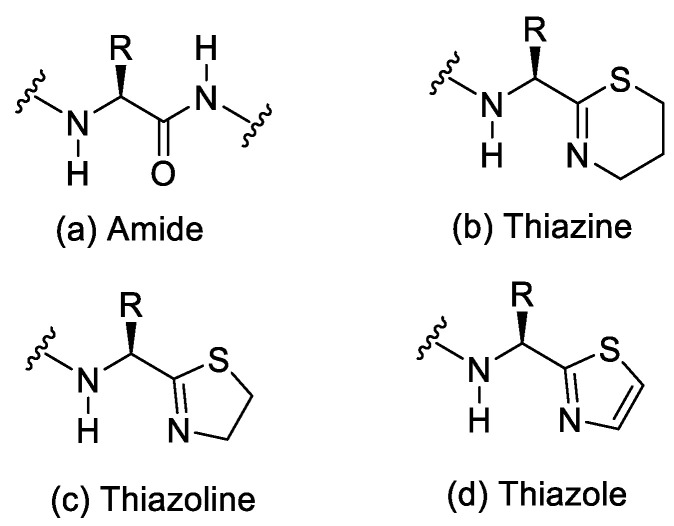
Comparison of the geometries at amide (**a**), 2-substituted 1,3-thiazine (**b**), 1,3-thiazoline (**c**), and 1,3-thiazole (**d**).

Tumminakatti et al. (2014) reported racemisation in a study of the synthesis of mono- and oligopeptidomimics with Thi modification at the C-terminus, derived from corresponding N-(3-hydroxypropyl) thioamide derivatives (Figure 17) [42].

**Figure 18:**
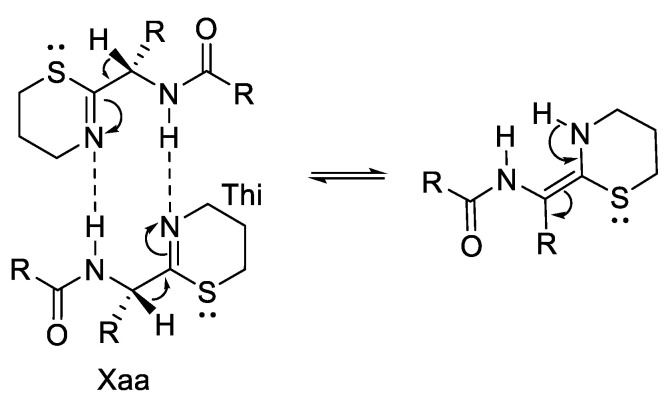
Possible intermolecular H-bond-assisted enamination/racemisation mechanism in non-Pro*-Thi containing analogues.

From Table 6, we can see that racemisation occurred in the Phe-Thi-containing peptide, which is shown in two sets of resonances with comparable intensities of ~1:1 in ^1^H and ^13^C NMR. The epimerisation product is not observed in the corresponding Pro*-Thi containing 2a (Table 7), which shows only a single set of resonances. The racemisation mechanism in non-Pro*-Thi containing analogues can be seen in Figure 18.

### 2.3. Bases

#### 2.3.1. Bases Used in Amino Acid Activation

**Figure 19:**
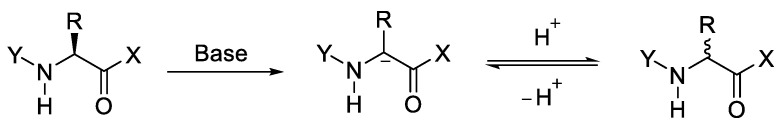
Mechanism of racemisation through alpha hydrogen attack.

In peptide synthesis, a tertiary base is usually used. Structurally, the base has a fairly high steric hindrance and, as a result, the nucleophilicity of the base decreases so that the attack of alpha hydrogen can be suppressed [43]. On the other hand, the addition of a base that has little steric hindrance opens up opportunities for an alpha hydrogen attack, which can cause racemisation. The mechanism of racemisation caused by bases can be seen in Figure 19.

Epimerisation of a peptide molecule in nature can be catalysed using racemase and epimerase enzymes [3]. This enzyme requires one or two steps to abstract the α-H proton from an amino acid in a protein or peptide through a deprotonation/reprotonation mechanism. Research conducted by Jadhav et al. (2021) mentioned that potassium ethoxide can be used for proton abstraction from one side. Meanwhile, protonation by the solvent occurs through the other side to produce D-amino acid [5].

Another experiment was conducted where a milder base 2,4,6-collidine (entry 2) was used. A low conversion was observed, whereas a high degree of epimerisation was detected. The combination of DIEA as base and DMF as solvent gave the highest conversion and the lowest epimerisation percentage (13%, entry 3).

In other study, Steinauer et al. (1989) reported the effect of the base in the rate of racemisation associated with the use of benzotriazol-1-yl tris(dimethy1amino)phosphonium hexafluorophosphate (BOP) [44].

**Figure 20:**
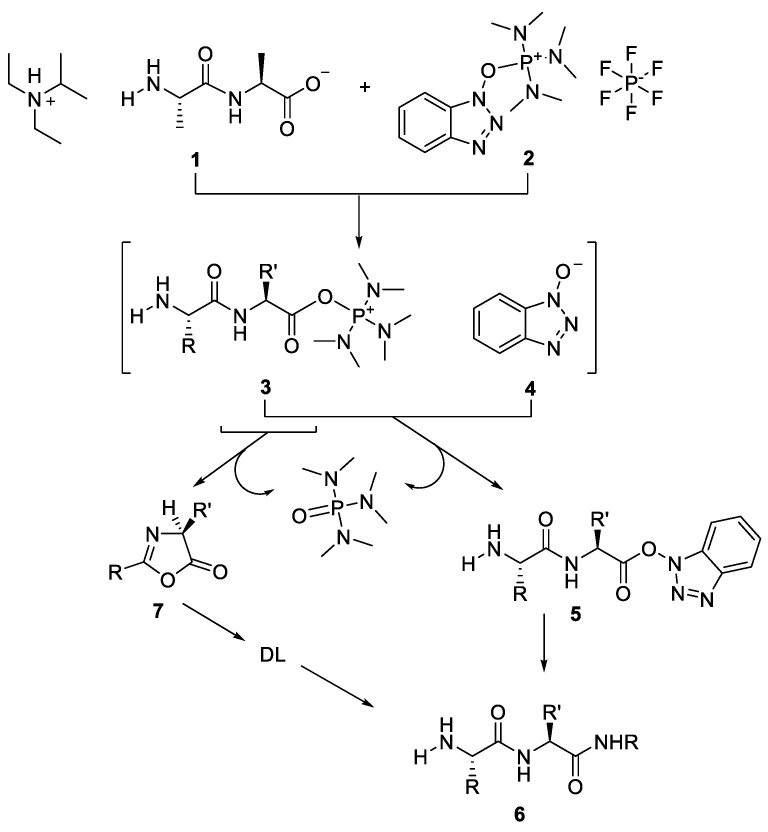
Racemisation process in BOP activation with the effect of base [45].

Figure 20 demonstrated that the formation of anion **1** by the addition of a tertiary amine is the first step in the activation of amino acids utilising a BOP coupling reagent. Following that, the reaction between the carboxylate anion **1** and the reagent 2’s phosphorus atom resulted in the formation of the acylphosphonium cation **3** and its counterion, the benzotriazolyl anion **4**. After that, the anion **4** attacked the carbonyl of **3** to create the benzotriazolyl ester **5**, which underwent aminolysis to create the peptide **6**. Racemisation most likely occurs just before anion **4** attacks, at which point the acylphosphonium cation **3** undergoes cyclisation to form the chirally Unstable 2,4-disubstituted-5(4H)-oxazolone 7. The kind and quantity of the tertiary amines employed in the BOP-mediated couplings of the model peptides Z-GlyXxx-OH with H-Val-OEt were investigated by the author. By using high-performance liquid chromatography to identify the epimeric products of the reactions, the extents of racemisation were discovered (16). Table 8 shows the reaction results in dichloromethane.

From Table 8, it can be concluded that the racemisation occurred in all the reactions examined. DIPEA led to the least racemisation, meaning that the amount of racemisation varied inversely with the extent of hindrance about the nitrogen atom of the base. The stereomutation increased dramatically in the presence of a third equivalent of a base. Much less racemisation occurred when a deficiency of base had been added, but the yields were low.

#### 2.3.2. Bases Used in Fmoc Deprotection

**Figure 21:**
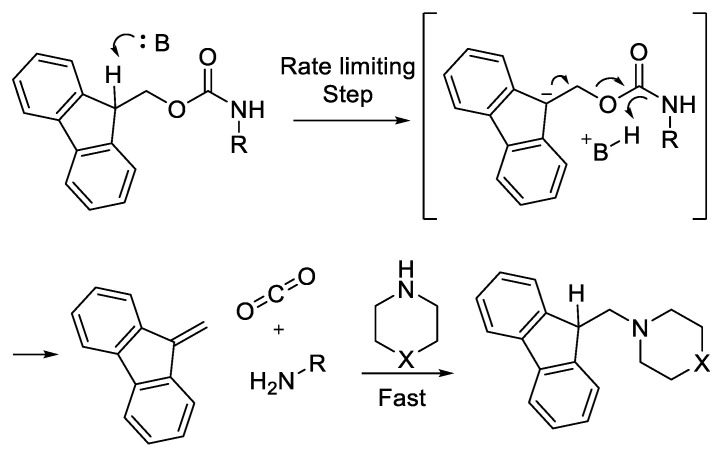
Base-induced cleavage of Fmoc and subsequent quenching of dibenzofulvene by a nucleophile.

Apart from being used in amino acid activation, bases are also used in Fmoc deprotection [46]. Fmoc-deprotection in SPPS is undertaken by an unhindered nucleophilic base, such as piperidine in polar solvents, while DMF proceeds through a two-step mechanism. The first step is the removal of the Fmoc group, followed by quenching of the resultant by-product dibenzofulvene (DBF). The detachment of Fmoc is through a E1CB mechanism with the abstraction of protons from the bulky 9-fluorenylmethyl by the bulky base, which is the rate-determining step (Figure 21). The excess of DBF by-product is a highly reactive electrophile [47].

Several studies investigated the contribution of Fmoc deprotection step to the racemisation of amino acid during the SPPS method. Ralhan et al. (2015) reported the effect of deprotection solutions on levels of epimerisation in resin that bound model tripeptide GCF (Table 9) [48].

**Figure 22:**
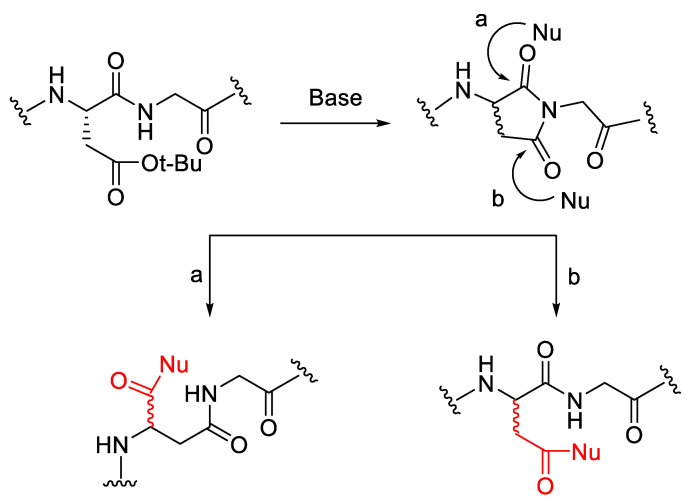
Epimerisation via the aspartimide mechanism in the deprotection step.

As shown in Table 10, there are no significant effects from the addition of DBU and the epimerisation rate of Fmoc deprotection. The different approaches have been reported to analyse the problem of aspartimide formation in Fmoc/^t^Bu-based SPPS, such as using a sterically hindered side chain protection, including OMpe and OEpe. The most common side reaction in Fmoc-SPPS, which is induced by the base, is the formation of aspartimide, which occurs due to the attack of the amide nitrogen on the carbonyl part of the protected aspartic acid side chain to form aminosucinate [49]. The imide ring can be opened by nucleophile attacks, such as from piperidine, piperazine, or residual water, and form α-aspartyl and β-aspartyl peptides 2 [14] (Figure 22). 

Ralhan et al. (2015) further synthesised VKDGYI, a hexapeptide originally derived from toxin II of the scorpion *Androctonusaustralis hector*, which has an aspartatic acid residue, to test the compatibility of deprotection solution. We observed 1% formic acid (FA), as the acid was chosen as an additive, and the deprotection solution of 5% piperazine, 1% DBU, and 1% FA was modified to effectively mitigate the formation of aspartimide [48].

Table 10 shows that the use of piperidine as a deprotection cocktail results in a high degree of aspartimide formation. The addition of FA lowered aspartimide formation and led to higher recovery of target peptide compared to no FA solutions. The mixture of 5% piperazine + 1% DBU + 1% FA caused lower aspartimide formation than 20% piperidine + 1% FA. From these data, we can conclude that FA-modified piperazine/DBU solution can suppress the yield of aspartimide side reactions for most SPPS applications.

### 2.4. Steric Factor and Ring Orientation

Jadhav et al. (2021) reported that the peptide with an eight-membered ring was more susceptible to epimerisation compared to the nine-membered peptide. This phenomenon was caused by steric factors, which potentially affect the abstraction of alpha protons (Table 11). The result provided in Table 12 shows that a cyclisation with higher steric factor (ring 8) causes higher epimerisation than the lower steric factor (ring 9) [5].

**Figure 23:**
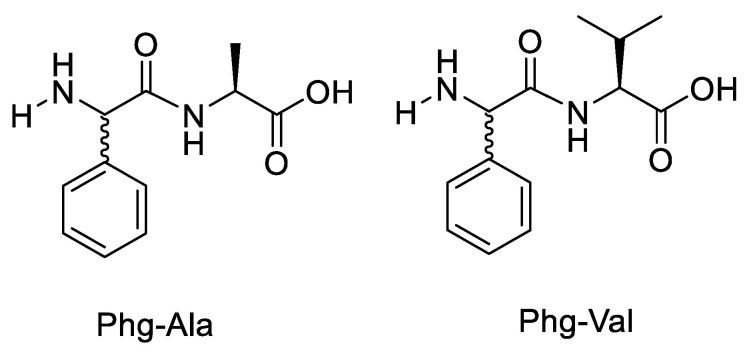
Structure of dipeptide Phg-Ala and Phg-Val.

In another work, pyridoxal was used as a catalyst in the epimerisation of N-terminal peptide residues by Danger et al. (2010) to evaluate the effect of steric hindrance of the C-terminal residue in dipeptides [49]. Since amino acids with aryl side chains epimerise more quickly, phenylglycine (Phg) residues near the N-terminus of peptides were particularly chosen (Figure 23) [50]. Phg’s side chain was chosen to enable the experimental study and was designed to function similarly to the side chains of other amino acids. To evaluate the impact of sterical hindrance on epimerisation, the dipeptides with phenylglycine as an N-terminal residue and Ala or Val as a C-terminal residue were compared.

Since the Diastereoselective Excesses (deL) are enhanced in the presence of Val residue, as shown by the value obtained with Val dipeptide for which deL = −42% compared to the one obtained for Ala dipeptide for which deL = −13% (Table 12), this stabilisation seems to be increased by the hindrance of the neighbouring residue. The rate of development of deL for Phg-Val is approximately two times slower than that for Phg-Ala at the same time, with the kinetic constants for epimerisation (krDL and krLL) being equal to 6.5 × 10^−^^6^ s^−^^1^ and 12.4 × 10^−^^6^ s^−^^1^, respectively.

**Figure 24:**
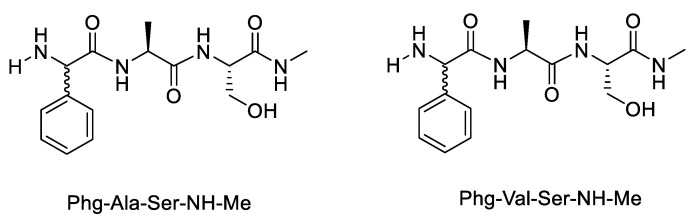
Phenylglycine-containing peptide.

Danger et al. also observed the effect of the length of the peptide chain. To detect a possible effect of the length of peptide chains, a third residue was added at their C-terminal position. Thus, L- or D-Phg-L-Xaa-L-Ser-NH-Me tripeptides (Figure 24) were chosen.

The thermodynamic equilibrium deL for peptides containing Ala changes from −13% for dipeptides to −1% for tripeptides (Table 12). In the addition of a Val residue, this relative stabilisation of homochiral structures was considerably more important because deL increases from −42% for dipeptides to −16% for tripeptides. As a result, the stability gap between epimers was reduced by the inclusion of a third residue, and this effect was more pronounced when residues were sterically inhibited. The high diastereoisomeric excess, however, is not supported by either of the results obtained with these Phg-containing model peptides, which suggests that short peptides are unlikely to experience symmetry breaking unless a different mechanism is found.

### 2.5. Solvent

Since we know that all racemisation processes proceed via a charged intermediate, during the epimerisation process the solvent also plays a major role in stabilising the resonance of the carbanion intermediate produced by the base, allowing the base to abstract protons. From this phenomenon we can see that the choice of organic solvent for peptide synthesis has a significant impact on the outcome of amino acid racemisation. However, the use of solvents in peptide synthesis, especially in solid-phase peptide synthesis, was limited by the swelling value of the resin used [51].

**Figure 25:**
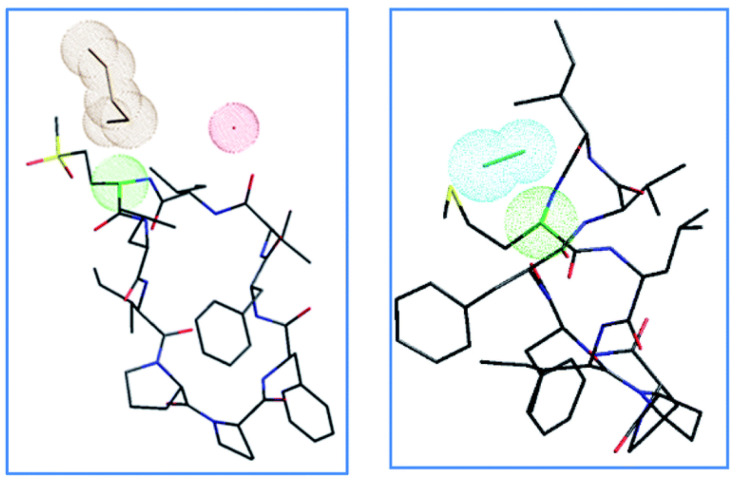
Crystal structures of LO4 butanol solvate and LO2 methanol solvate, with color-coded atoms sulphur—yellow, oxygen—red, nitrogen—blue, carbon—black, a carbon Met/MetO2—green; solvent clouds: butanol—brown, water—red, methanol—cyan [44].

For example, in the crystal structure of orbitide compounds in Figure 25, the solvent can play a significant role in the resonance stability of the carbanion intermediate formed by the base, permitting base access for proton abstraction. There have also been reports of an oxazolone mechanism supporting the influence of the sidechain in Met epimerisation. The presence of a solvent in the Met and its oxidised products structure may help to stabilise isomerisation intermediates [52].

### 2.6. Temperature

**Figure 26:**
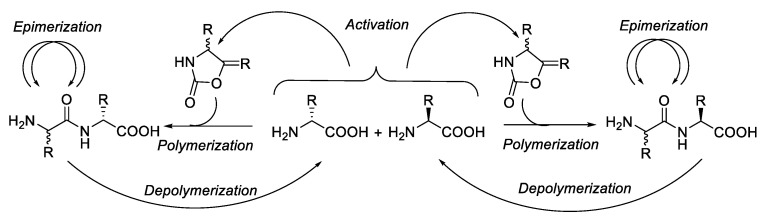
A representation of the APED model that is based on the Primary Pump Scenario and displays the antagonism between two chemical cycles that potentially induces dynamically controlled states such that *D*- or *L*-amino acids can prevail as a result of the reproduction of chirality through dipeptide epimerisation.

Danger et al. (2010) observed that epimerisation can occur at a temperature of 100 °C and in neutral pH. The activation of amino acid via N-carbamoylamino acids (CAAs) can lead to forming N-carboxyanhydrides (NCAs). The hydrolysis of NCA compounds at 100 °C between two chemical cycles that potentially induce dynamically controlled states such as that of *D*- or *L*-amino acids can prevail as a result of the reproduction of chirality through dipeptide epimerisation (Figure 26) [52].

### 2.7. Epimerisation in a Peptide Cyclisation

Cyclic peptides are small molecules that contain 5–30 amino acids that have many bioactivities [53]. For example, tyrocidine [54] and gramicidin [55] are two cyclopeptides used as antibiotics, and cyclosporine is used as an immunosuppressant drug. Cyclopeptides are more stable than linear peptides and resistant to protease degradation, and the cyclopeptide Gαi [NaC-(MITWYEFVAGTK)] in particular has shown good proteolytic instability compared to the parent linear molecule [56].

**Figure 27:**
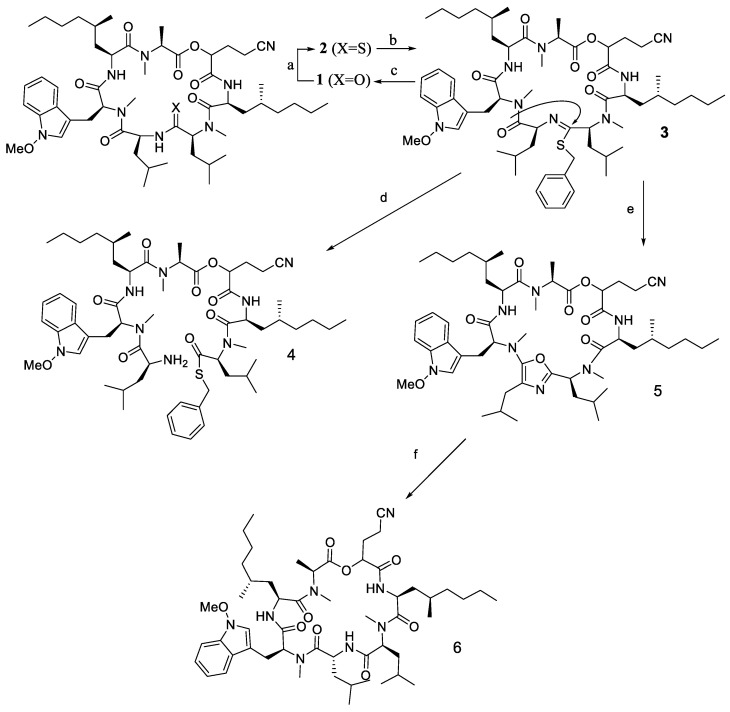
(**a**) Lawesson’s reagent, xylene, 130 °C, 30 min, 33%, (**b**) PhCH_2_Br, aq. NaOH/CH_2_Cl_2_, sonication, r.t., 30 min, 94%, (**c**) AgNO_3_, ^t^BuOH/water 9:1, r.t., 2.5 h, 35%, (**d**) aq.HCl/^t^BuOH, 55 °C, 20 min, quant, (**e**) HgCl_2_/CaCO_3_, AcCN, r.t., 2 h, 45%, (**f**) TFA, ^t^BuOH/water, 15 h, r.t., 35%.

However, the information of chemical synthesis for selective epimerisation of cyclic peptides has rarely been reported. One of the studies explaining epimerisation in cyclisation was conducted by Sikandar et al. [57]. After **1** underwent regioselective thionation at one of its six amide-bonds, epimerisation took place during the cyclisation process. The thioamide **2** was subsequently S-benzylated to produce the thioimidate **3**, which underwent a unique intramolecular cyclisation that was supported by mercury to produce the peptide-bridged 5-aminooxazole **5** (Figure 27). Oberhauser et al. (1999) also discovered that the L-Leu epimer (**4**) rather than the D-Leu epimer (**6**) was formed preferentially as a result of the two-step process using the achiral oxazole intermediate. When the chirality at Leu was destroyed by aromatisation, an unusually formed peptide-bridged 5-aminooxazole resulted [58].

**Figure 28:**
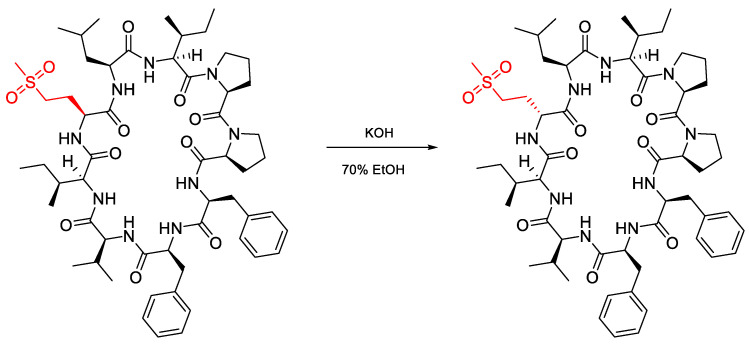
Changing the configuration of methionine from L to D using KOH base catalyst.

Jadhav et al. (2021) studied methionine epimerisation in cyclic peptides and found that cyclic peptides containing methionine either singularly or in oxidation form can undergo epimerisation. Methionine-containing cyclic peptide was oxidised using KOH in 70% EtOH, resulting in an epimerisation product from the cyclopeptide (Figure 28).

Based on Table 13, it can be seen that a compound containing unoxidised methionine has a smaller percentage of epimerisation (5.14%). Meanwhile, a compound that was oxidised with alkaline had a higher percentage of epimerisation (29.43%). However, a compound that is oxidised with alkaline in SO_2_ form results in a smaller percentage of epimerisation (20.48%).

Another study of epimerisation in cyclopeptide synthesis was conducted by Muhajir et al. (2021) and involved the synthesis of nocardiotide A using a combination of solid and solution phase methods [59]. Nocardiotide A is a cyclohexapeptide which has been isolated from *Nocardiopsis* sp. bacteria in the marine sponge *Callyspongia* sp., and has the potential activity to affect several cancer cells, specifically against MM.1S multiple myeloma, Human HeLa cervix carcinoma, and Murine CT26 colon carcinoma cells with IC_50_ of 8, 11, and 12 µM/mL, respectively. The synthesis of the linear precursor of nocardiotide A was undertaken easily using the solid-phase method, with HBTU/HOBt acting as a coupling agent in the presence of basic DIPEA. The synthetic issue occurred during the macrocyclisation process in the solution phase.

**Figure 29:**
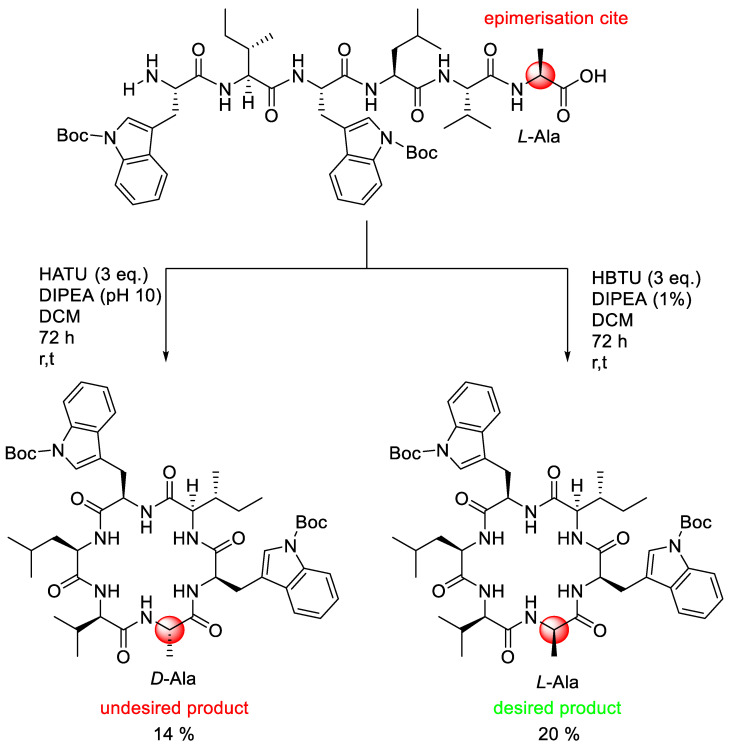
Epimerisation product in cyclisation step nocardiotide A.

From Figure 29, it can be seen that the cyclisation was undertaken using a head-to-tail strategy where alanine residue was chosen as the C-terminal and tryptophan as the N-terminal. In this case, cyclisation of linear peptide using HATU coupling reagent underwent racemisation on the alanine residue. The racemisation was shown in the difference in the ^13^C- and ^1^H-NMR chemical shift between the synthesis and the isolation product. Fortunately, the racemisation product was not observed when the coupling agent changed into the HBTU coupling agent. The yield of the cyclisation product is also increased in the presence of 14% HATU and 20% HBTU coupling agent.

## 3. Suppressing Epimerisation

### 3.1. Coupling Agent Selection

Amide bond formation is a key process in the synthesis of peptides. The many studies conducted have shown that it is important to provide the selection of coupling agents. The activation of carboxylic acid into active ester is a potential step to forming oxazolone and leading to an epimerisation product (Figure 4). Generally, carbodiimide-based reagents combine strong acylation potency and smooth reaction conditions, and they are commonly used in the presence of additives. Several studies reported the effectiveness of the coupling agent and its addition in several amino acid activations [60].

**Figure 30:**
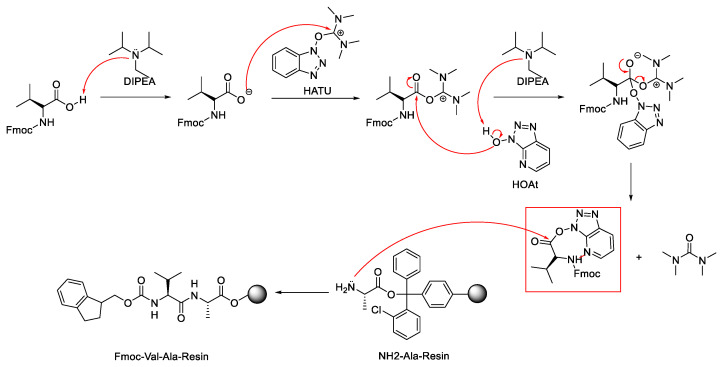
Mechanism of coupling reaction facilitated by coupling reagent HATU/HOAt.

Figure 30 shows the mechanism of the coupling reaction using a combination of uranium-based reagent (HATU) and its additive, HOAt [59]. Initially, the amino acid deprotonated by DIPEA reacts with HATU, producing activated acyluronium species and an oxy-7-azabenzotriazole (OAt-) anion. The OAt- anion immediately reacts with the activated acid to produce a reactive ester, which then undergoes aminolysis. In this reaction, a by-product of insoluble urea is produced.

The nitrogen atom in oxy-7-azabenzotriazole (OAt-) ester induces hydrogen binding with the N proton, covering the formation of oxazolone from the Fmoc protecting group and suppressing the epimerisation product. Within this phenomenon, the HATU/HOAt coupling agent was widely used in amide formation in solid- and solution-phase peptide synthesis.

**Figure 31:**
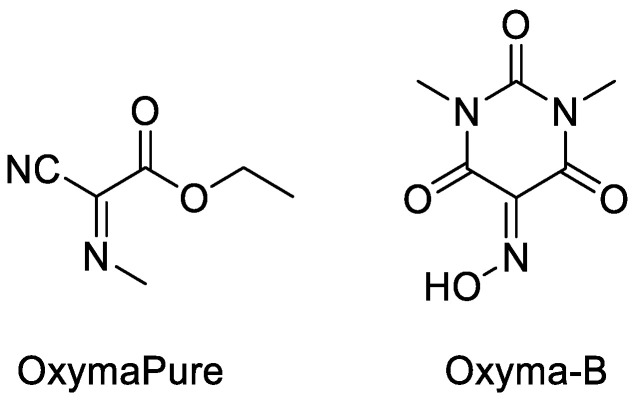
The difference in structure between OxymaPure and Oxyma-B.

Jadav et al. (2014) reported a new oxime additive, 5-(hydroxyimino)-1,3-dimethylpyrimidine-2,4,6(1H,3H,5H)-trione (Oxyma-B) (Figure 31), which was more effective in the control of optical purity during the synthesis in the carbodiimide acylation process. The coupling condition was tested in several amino acids that have a high potency of epimerisation products, such as histidine, cysteine, and serine in tripeptide H-Gly-AA-Phe-NH_2_ form [5].

As can be seen from the evaluation result in Table 14, Oxyma-B 14 stands out as one of the additives for peptide synthesis that has the greatest potential. When used as a racemisation suppressor, it demonstrated superior performance than OxymaPure 7 and outperformed HOAt 2 in both stepwise and segment coupling in solid- and solution-phase peptide synthesis.

**Figure 32:**
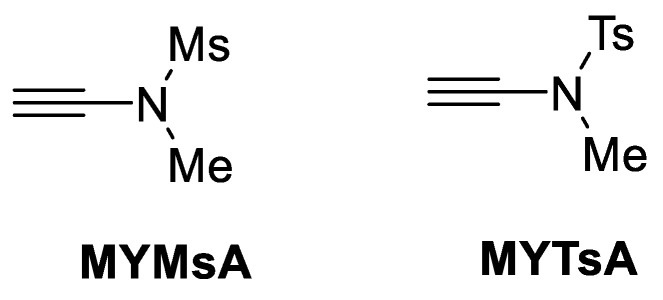
Structure of ynamides (MSMsA and MYTsA).

Ynamides were created in another investigation as novel coupling reagents under incredibly benign reaction conditions [61]. Ynamides feature an electron-withdrawing group (EWG) on the nitrogen atom, which makes them a stable substance that is simple to synthesise and manage. The danger of base-induced racemisation can be suppressed by ynamides because they are less basic or nearly neutral (Figure 32) [62].

Several coupling agents were tested to compare the epimerisation/racemisation product in amide bond formation. Table 15 shows the coupling of H-L-Leu-O^t^Bu and Fmoc-Ser(^t^Bu)-OH, which has high potential epimerisation, and this epimerisation was analysed using HPLC analysis. The significant epimerisation product occurred with conventional coupling reagents such as HBTU, HATU, PYBOp, and DCC. The DEPBT coupling reagent produced a low epimerisation product but also reduced the reaction efficiency. The ynamide coupling reagent of both MYMsA and MYMTsA yielded good efficiency and low epimerisation product in amide bond formations. From this result, it can be assumed that ynamides could be a practical coupling reagent for amide and peptide synthesis in both academia and industry settings.

The dehydrative peptide bond forms of carboxylic-amino acids with a free hydroxy group in the -position were reported by Koshizuka et al. (2020) in another study [63]. Low catalyst loading during this hydroxy-directed reaction allowed for the direct synthesis of dipeptides derived from serine or threonine with great chemo selectivity, low epimerisation, and high functional group tolerance [64,65].

To explore the effectiveness of DBAA-catalysed dehydrative peptide bond formation in the suppression of the epimerisation, DBAA was tested to catalyse several amino acid couplings with a different protecting group and amino acid side chain. As serine N-protecting groups, Cbz, Boc, and Fmoc could be employed. All of the reactions in Table 16 involving the amino acids H-Ala-O^t^Bu, H-Leu-OMe, H-Ile-OMe, and H-Val-OMe that had an alkyl side chain at the α-position were successful within 24 h, and dipeptides (entry 1–6) were produced in high to excellent yields (74 > 99%). Additionally, unprotected H-Tyr-OMe produced substantial yields of the dipeptide Boc-Ser-TyrOMe. Thus, it appears that a phenolic hydroxy group that is not protected does not prevent the current catalysis from occurring. H-Asp(^t^Bu)-O^t^Bu, a derivative of aspartic acid, yielded dipeptide in 97% of the cases. The similar outcome was demonstrated for dipeptides (entry 15–23) provided by H-Trp-OMe, H-Lys(Boc)-OMe (3p), H-Arg(Pbf)-OMe, and H-His(Trt)-OMe in respectable yields (64–99%). It is noteworthy that stereochemical integrity was almost always preserved (dr 96/4 > 99/1). This finding indicates that DBAA is a potent catalyst for the hydroxy-directed amidation of a wide variety of β-hydroxy-α-amino acids with α-amino esters, resulting in dipeptides with high yields and a low level of epimerisation.

### 3.2. Side Chain Protection Group

**Figure 33:**
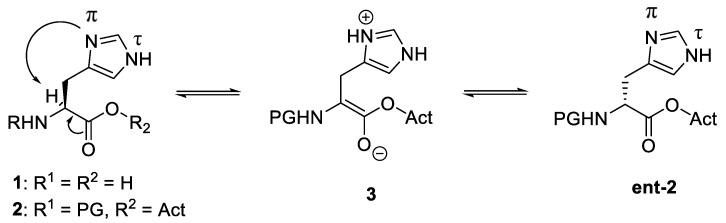
Epimerisation mechanism in histidine amino acid residue.

One of the most potential racemisation-centric amino acids is histidine, as it contains a basic imidazole moiety with two nitrogen atoms. However, the basicity of the imidazole moiety causes difficulties in peptide synthesis [66]. Specifically, the His residue exhibits a high propensity toward epimerisation, especially when its carboxylic moiety is activated in acid halides or acid anhydrides (Figure 33).

Figure 33 shows that N(π) atom’s attempted abstraction of H from C to produce enolate 3, which provides L-His derivative 2 and its enantiomer ent-2 at the same rate, initiates the epimerisation. It was originally tried to diminish the basicity of the N(τ) atom indirectly by protecting the more nucleophilic, and hence more readily and selectively protectable, N(π) atom with bulky and/or electron-withdrawing groups such as triphenylmethyl (Trt). However, given the existence of the free N(τ) atom, which is thought to work directly as a base [67], this N(π) protection only significantly inhibited the racemisation.

**Figure 34:**
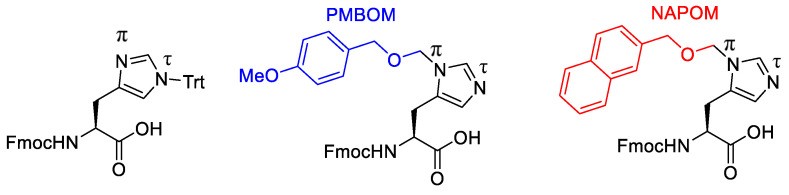
Structures of protected histidine.

Torikai et al. (2020) reported the development of the histidine side chain protecting group, especially on the N(τ) atoms, such as p-methoxybenzyloxymethyl (PMBOM) and 2-naphthylmethoxymethyl (NAPOM) (Figure 34). PMBOM actually has a good effect on suppressing the epimerisation of histidine. However, the generation of PMBOM had issues due to the requirement for low-temperature reactions and chromatographic purification during the synthesis of PMBOMCl. Later, the (NAPOM) protection group served as an alternative and is also orthogonal to PMB(OM), which is more easily generated than PMBOM [68].

Another study reported a strategy to suppress the epimerisation potential of Cys amino acid caused by basic treatment in side chain protecting groups. When the initial Cys was attached to the resin, the epimerisation from L-Cys to D-Cys was optimised to neatly afford Fmoc-Cys(PG)-O-resin (PG: any protective group). During peptide elongation, these adverse events reduced the quality of the peptide. Because it frequently co-elutes with the desired target peptide, the undesirable C-terminal D-Cys-containing peptide in particular is frequently undetected by HPLC [69].

### 3.3. Coupling Strategy

#### 3.3.1. Pseudoproline

**Figure 35:**
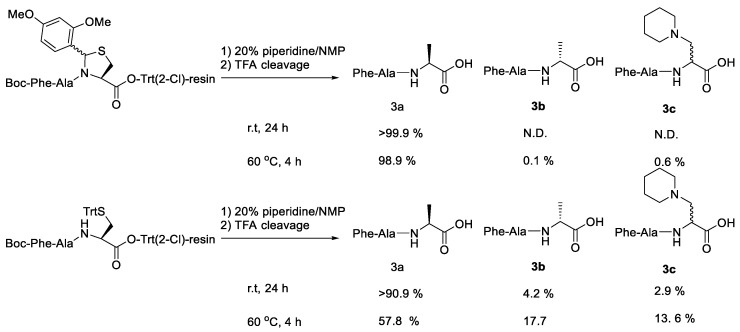
Comparison study of the epimerisation rate at C-Terminal Cys of protected peptide acid on the resin.

Using a C-terminal pseudoproline protecting group and Trt-type resin, Tsuda et al. (2020) showed practically how to suppress the side reactions (Figure 35. Under standard/heated basic conditions, a comparison of the side reaction rates of the C-terminal Cys(Trt)-O-Trt(2-Cl)-resin and the C-terminal pseudoproline-O-Trt(2-Cl)-resin showed that the latter is significantly better at suppressing the side reactions. Additionally, epimerisation-free segment condensation processes using the protected peptide segment with the C-terminal pseudoproline structure produced by this technique were successful [70].

By creating Boc-PheAla-Cys(Dmp,Hpro)-O-Trt(2-Cl)-resin (**1**) and Boc-Phe-AlaCys(Trt)-O-Trt(2-Cl)-resin (**2**), the epimerisation rate of the C-terminal Cys of the protected peptide acid on the resin was compared. Without any issues, the Cys-pseudoproline dipeptide was added to the Cl-Trt(2-Cl)-resin in the presence of DIEA, and subsequently Boc-PheOH was added to the resin. The Fmoc protecting group was then deprotected utilising fundamental conditions (20% piperidine in NMP) over the course of 24 h at room temperature (Figure 35). Following TFA-mediated deprotection, HPLC was used to track the impurity formation. Therefore, when Cys(ΨDmp, Hpro) was used, there was no identifiable side product resulting in the crude. However, when Cys(Trt) was used, D-Cys-containing peptide (3b, 4.2%) and D/L-piperidinoalanine-containing peptides (3c, 2.0 and 2.9%, respectively) were found. The epimerisation product of cysteine amino acid can be suppressed by the selection of the pseudoproline-type protecting group, according to these findings. 

#### 3.3.2. Copper(II)-Mediated Chan–Lam-Type Coupling

**Figure 36:**
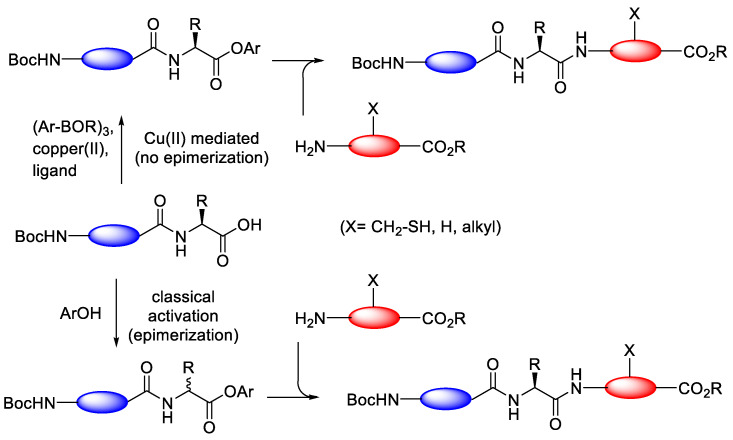
Copper(II)-mediated and classical peptide arylester synthesis and subsequent elongation.

Another strategy was also developed to enhance the racemisation free C-terminal peptide activation through the copper(II)-mediated Chan–Lam-type coupling between peptides and arylboroxines and subsequent amine-coupling reactions [71]. Recently, the used of Cu(OTf)_2_-mediated reaction of benzoic acids with arylboronic acids was reported to provide facile access to arylester [72]. One example of this feature is in the coupling of phenylacetic acid, which can combine efficiently with phenylboronic acid to soon give an ester. From these results, it is possible to extend this chemistry towards the peptide synthesis [73]. The pathway mechanism of this reaction and the mild conditions indicate that epimerisation can be suppressed (Figure 36).

Popovic et al. (2013) reported the effectiveness of free racemisation of the Chan–Lam-type esterification of peptide by activating several amino acids using phenylboronic acid (3 equiv.), Cu(OTf)_2_ (0.4 equiv.), and urea (1 equiv.) at elevated temperature (65 °C) under air using EtOAc as the solvent [74].

Table 17 demonstrates that under optimal conditions, Cu(OTf)_2_-mediated coupling of Boc-Trp-Phe-OH with 4-(methylthio)phenyl boroxine produced the ester in an isolated yield of 57% (Table 17, entry 1). This is the study’s lower yield, which was attained. With mCPBA acting as the oxidising agent, a quantifiable yield was achieved during the partial oxidation of the thioester to the sulfoxide. However, it proved impossible to further oxidise the sulfone without also damaging the tryptophan indole moiety. Fortunately, employing oxone as the oxidising agent, the synthesis of Boc-Phe-Phe-4 (methylsulfonyl)phenyl ester was successful, with a 77% total yield (entry 2). The remaining material was then simply removed using non-basic water extraction. The other esterification resulted in a very substantial diastereomer excess (entry 2–9), demonstrating the racemisation-suppressing ability of the Cu(OTf)_2_-mediated coupling peptide.

Another study also indicated the role of cupper in suppressing epimerisation instead of playing a role in a Copper(II)-mediated Chan–Lam reaction [75]. Several studies have employed metal salt to find more suppressant additives for epimerisation-free peptide coupling (such as AlCl_3_, ZnCl_2_, SbCl_3_, and CuCl_2_), and other inorganic salts in the carbodiimide method prevent epimerisation [76]. The Cu^2+^ ion was found to have the lowest levels of epimerisation (D-epimer, 0.1%) as an epimerisation suppressant additive in several amino acid coupling methods [77].

Ryadnov et al. (1999) observed the use of any additives to suppress epimerisation and evaluated the role of Cu^2+^ in reducing the epimerisation product [78]. From Table 18, it can be seen that inorganic salt, such as ZnCl_2_, RbClO_4_, SnCl_4_, AlCl_3_, and BF_3,_ can obtain couplings with minimal epimerisation (D-epimer, 1%) in an additive with the carboxy component (1:1). Unfortunately, in another study, these inorganic salts varied depending on the activation time and coupling of the extent of epimerisation [79]. The other inorganic salts showed high epimerisation products in the process of coupling. It can be seen that only CuCl_2_ salt can influence epimerisation and appeared to yield a standard result (D-epimer, 0.1%). This result is independent of both equivalent quantities and activation duration. Thus, from this result, it can be concluded that Cu^2+^ is an effective additive to achieve epimerisation-free peptide coupling.

#### 3.3.3. Hydrosilane-Mediated Type Coupling

Racemisation via direct enolisation or formation of the corresponding oxazolone intermediates during elongation still seems to be the highest potency point in peptide synthesis [80]. To suppress these issues, additives such as HOBt and Oxyma, and their derived peptide coupling reagents, and other emerging coupling reagents, such as T3P and DEPBT, which certainly show remarkable resistance to racemisation, have been developed [81,82]. However, beyond the several allergic reactions seen with HATU, they also have explosive properties like those of HOBt. Moreover, T_3_P and DEPBT have a high price, thus meaning they are used in peptide synthesis and racemisation, and are still problematic in SPPS [83]. Because of increasing interest in peptide therapeutics over the past few decades, technologies that can suppress the racemisation issue as well as waste production, and alternatives to these harmful reagents, have been developed in recent years [79].

**Figure 37:**
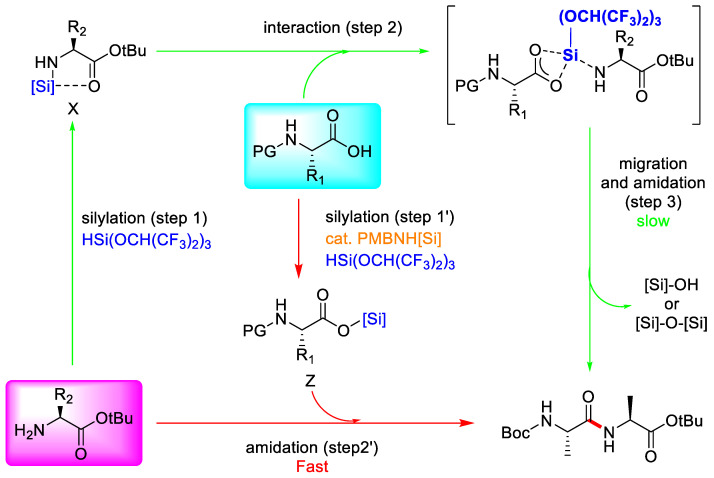
Epimerisation-free mechanism of 3 hydrosilane-mediated type coupling.

Muramatsu et al. (2020) developed a new strategy of peptide bond forming via hydrosilane-mediated amino acid silyl esters, which offer great epimerisation-free features, including high efficiency, broad scope, strong functional group tolerance, being metal-free and cost-effective, harmless by-products, and sustainable procedures [84]. The silyl agent can interact with other amino acids, which can block the way of epimerisation via an oxazolone process (Figure 37). 

From Table 19, it can be seen that several silylating reagents were evaluated for the construction of the peptide bond between Boc-L-Ala-OH and H-L-Ala-O^t^Bu to find the best silylating agent with high yield and diastereomer purity. In the first trial (entry 1), when Boc-L-Ala-OH was treated with 2 equivalents of H-L-Ala-O^t^Bu in the presence of 1 equivalent of (trimethylsilyl)imidazole (TMSIM) at room temperature, the silyl ester, which is generated in situ by the silylation of Boc-L-Ala-OH with TMSIM, it did not react with H-L-Ala-O^t^Bu to provide the desired dipeptide. The reaction occurred when the TMSIM was replaced by ClSi(OEt)_3_ (30% yield), but it took a long time to occur. The other silylating agent showed a high diastereomer purity, thus indicating that the interaction of silyl with amino acid can suppress the epimerisation product. In order to produce the desired dipeptide in 95% yield with no epimerisation, the amidation reaction of Boc-L-Ala-OH with 1.5 equiv of H-L-AlaO^t^Bu in the presence of HSi(OCH(CF_3_)_2_)_3_ in DCM at room temperature was shown to be the optimal scenario (entry 8). It is simple to make HSi(OCH(CF_3_)_2_)_3_ using trichlorosilane and 1,1,1,3,3,3-hexafluoro-2-propanol (HFIP). DCM can be replaced by a number of eco-friendly and popular industrial solvents, such as toluene. This finding leads us to the conclusion that, under mild reaction conditions, the hydrosilane-mediated reaction HSi(OCH(CF_3_)_2_)_3_ proceeds successfully with a broad functional group tolerance and without any degradation of stereochemical integrity.

#### 3.3.4. Native Chemical Ligation (NCL)

Total chemical synthesis of large peptides or proteins is one method that was widely used in drug research because of the easy access to modified proteins with high stability and improved biological activity, which can be key in understanding the importance of various post-translational modifications [85]. However, amino acid racemisation in the carboxylic activation step is still problematic during the elongation process in peptide synthesis, especially in synthesis of larger peptides or proteins. A convergent strategy was applied to minimise the side reaction of synthesising protein [86]. 1,2-Native Chemical Ligation (NCL) has offered a synthetic strategy for peptide segment condensation with respect to large peptide construction. This revolutionised synthetic strategy relies on the reaction of a peptide thioester with a cysteinyl peptide, and can be applied to the synthesis of peptide thioesters using Fmoc or Boc solid-phase peptide synthesis (SPPS) approaches [86].

**Figure 38:**
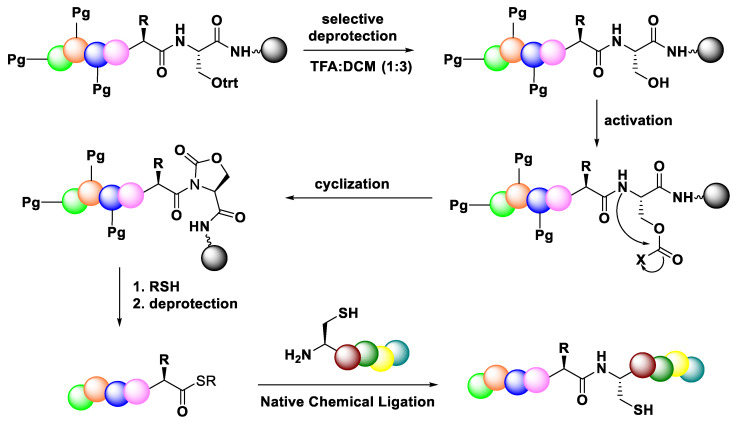
General steps for generation of peptide thioesters via Boc-based SPPS utilising special thioester linkers.

Figure 38 illustrates the typical method for producing peptide thioesters using Boc-based SPPS, which makes use of special thioester linkers. Fmoc SPPS is not applicable to thioester resins because peptide thioesters are unstable toward the piperidine treatment needed to deprotect the Fmoc group. The NCL strategy has several advantages in the synthesis of large peptides and protein. The reaction usually proceeds smoothly without major side reactions, and the reaction has proven to be highly chemical selective because the NCL reaction occurs between the side chain unprotected fragment. Moreover, this strategy does not need a carboxylic activation, which is the main cause of the epimerisation process.

**Figure 39:**
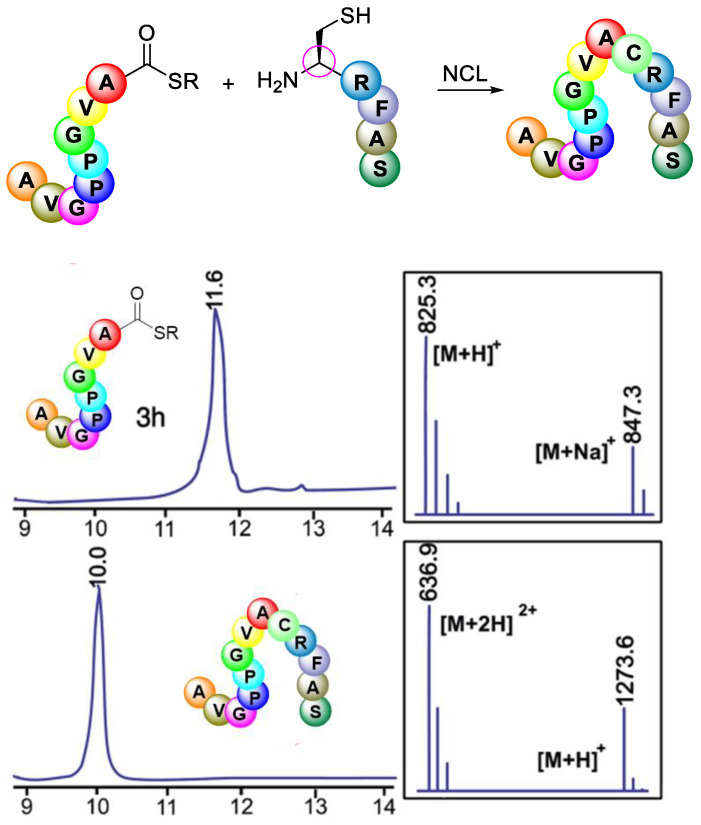
Native Chemical Ligation of peptide thioester Ac-AVGPPGVACOSR 3 h with N-terminal cysteine peptide CRFAS-NH_2_. HPLC/MS traces of peptide thioester 3 h and ligated product. SR ¼ S-(CH_2_)_2_-COOC_2_H_5_.

Elashal et al. (2016) examined Native Chemical Ligation by utilising peptide thioesters Ac-GPMLA-COS-(CH_2_)_2_-COOC2H5 3G and Ac-AVGPPGVA-COS-(CH_2_)_2_-COOC_2_H_5,_ which were previously generated by the cyclic urethane technique in Native Chemical Ligation (NCL) with N-terminal cysteine containing peptide CRFAS-NH_2_ (Figure 39). From Figure 39, it can be seen that the HPLC/MS spectra showed no epimerisation product in the NCL process, thus concluding that the NCL strategy can result in epimerisation-free peptide ligation [87].

**Figure 40:**
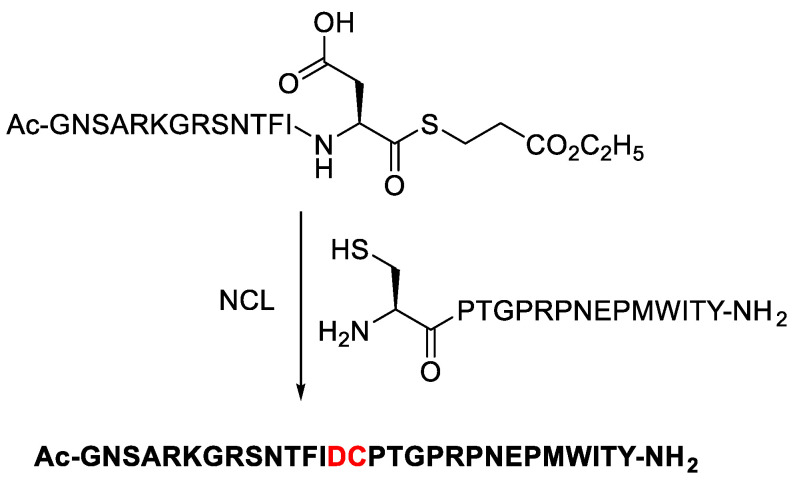

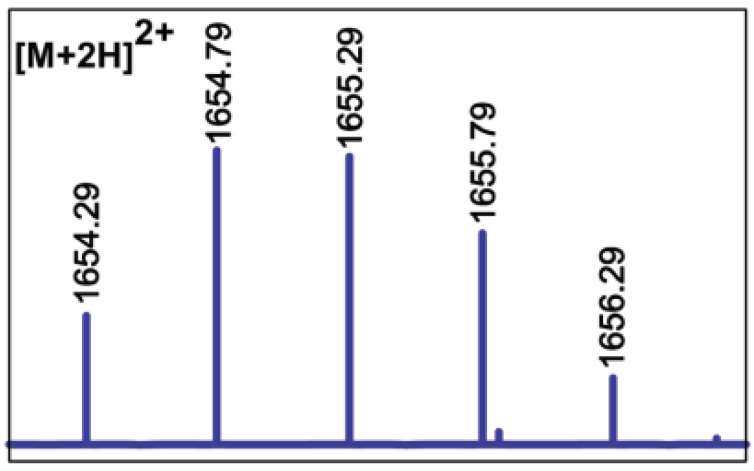
Ligation of peptide thioester Ac-GNSARKGRSNTFID-COSR 3Q with N-terminal cysteine peptide CPTGPRPNEPMWITY-NH_2_. MS of the ligated product. SR ¼ S-(CH_2_)_2_-COOC_2_H_5_.

A 29 amino acid-long peptide fragment of the rabies virus glycoprotein (Rvg), which was a successful carrier for cargo delivery into cells and for crossing the blood–brain barrier, was created by Elashal to ensure the success of the NCL method (Figure 40). AcGNSARKGRSNTFID-COSR 3Q, a 14 amino acid-long peptide thioester that was generated by the cyclic urethane method with a 39% yield, was ligated with the cysteine-containing peptide CPTGPRPNEPMWITYNH2. With very low concentrations of the peptide reactants (1.2 mM) and the 4-mercaptophenylacetic acid catalyst (25 mM), the ligation was completed in 24 h at 37 °C. This outcome demonstrates the effectiveness of the cyclic urethane method for producing peptide thioesters for the production of big peptides utilising NCL.

### 3.4. Hydrolisis Strategy of Peptide from Resin

Peptide cleavage from the resin is the last important step to obtaining the desirable peptide from the solid-phase peptide synthesis method. In the Fmoc strategy, peptide is usually hydrolysed or cleaved using an acid reagent to produce free C-terminal peptide. In some peptide synthesis, the hydrolysis or cleavage step yielded a potential epimerisation product that lowered the yield and made the purification more difficult.

One potential type of C-terminal epimerisation is C-terminal cysteine containing peptide. The C-terminal Cys carboxylates are the most present sequences of disulfide-rich bioactive peptides, such as somatostatin, conotoxins, and neocyclosimides [65]. These C-terminal Cys peptides have served as scaffold templates in many studies and research on the structural engineering of disulfide-rich bioactive peptides, which are highly important drug candidates (Kuan Hu et al., 2016). Furthermore, the C-terminal Cys residue of these peptides can form an intramolecular disulfide bond with the other internal Cys residues. This interaction can stabilise their bioactivity-related conformations [88]. However, cysteine in C-terminal residues can undergo the epimerisation product process due to the high acidity of the Cα proton of Cys esters [89]. Epimerisation at the C-terminal Cys can occur in two ways: in the ester bond forming reaction during the anchoring of the C-terminal Cys onto a solid and the repeated deprotection step with piperidine during Fmoc SPPS [90].

**Figure 41:**
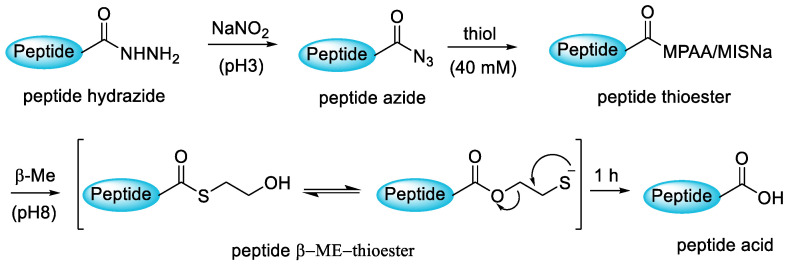
General route for the conversion of peptide hydrazide to peptide acid.

Zuo et al. (2019) developed a new way to synthesise the C-terminal cysteine containing peptide acids through a peptide hydrazide-based strategy. The strategy was carried out using unprotected Cys peptide hydrazides as precursors to synthesise C-terminal Cys peptide acids (Figure 41). The acidity of the Cα proton of the Cys hydrazide is much weaker than that of other ester groups, and thus the epimerisation and β-elimination at the C-terminal Cys can be effectively avoided [91].

**Figure 42:**
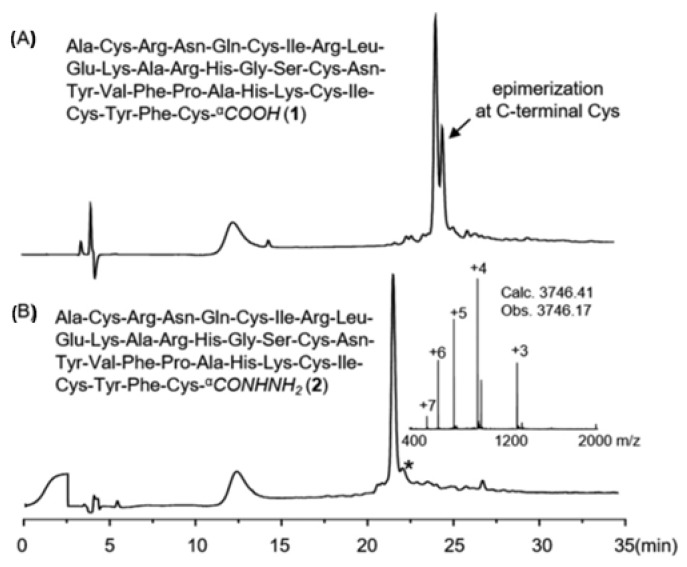
Analytical HPLC traces (210 nm) of (**A**) crude C-AhPDF 1.1b acid (**1**) using trityl(2-Cl) chloride resin and (**B**) crude C-AhPDF 1.1b hydrazide (**2**) using hydrazine-trityl(2-Cl) resin; * denotes the epimer of hydrazide 2.

Zuo et al. synthesised a C-terminal cysteine-containing peptide, namely C-AhPDF1.1b, via two different strategies. Firstly, C-AhPDF1.1b was synthetised using a 2-chlorotrityl resin through a microwave method. From Figure 42, it can be seen that the HPLC analysis of the crude C-AhPDF1.1b acid showed about 30% epimerisation at the C-terminal Cys site, making the purification step difficult. Gratifyingly, then C-AhPDF1.1b was synthetised using hydrazine-2-chlorotrityl resin. The epimerisation of C-AhPDF1.1b hydrazide was less than 3%. From this result, it can be concluded that the Fmoc SPPS of the C-Cys hydrazide peptide can suppress racemisation of the C-terminal Cys site. Additionally, the hydrazine-2-chlorotrityl resin was readily prepared via the hydrazinolysis of 2-chlorotrityl resin.

### 3.5. Ball Milling-Assisted

The chemical synthesis of lengthy peptides and proteins is extremely difficult in biological active peptide synthesis because coupling and deprotection reaction efficiency decline with peptide chain length, resulting in amino acid deletions and incomplete sequences. Peptide researchers have made significant efforts to overcome this difficulty, mostly by utilising convergent fragment coupling techniques, such as Native Chemical Ligation. The situation is further complicated by C-terminally activated peptide fragments, where a free N-terminal segment greatly increases the chance of epimerisation and produces very undesired diastereomers [92].

Yeboue et al. (2021) developed a new strategy to suppress the epimerisation product, by instead choosing an optimal coupling reagent and base. Yeboue et al. attempted a mechanochemical synthesis using solvent-free/solvent-less conditions. They considered analysing a ball mill’s ability to lessen or completely remove epimerisation during the C-terminal activation of peptide fragments and contrasting the outcomes with the traditional synthesis in solution [28].

Table 20 shows that Yebou et al. examined several coupling settings and identified two distinct reactions (magnetic stirring and vibration ball milling). High-performance liquid chromatography (HPLC) analysis of these data revealed that the tripeptide Z-Ala-Phg-Ile-OMe was extracted in a 93% yield, with exceptional purity and no traces of the Z-Ala-D-PhgIle-OMe epimer that could be detected (Table 1, entry 1; tR(LLL) = 5.08 min and tR(LDL) = 5.20 min). The temperature of the milled material increased somewhat during coupling and under ball milling circumstances; it reached 33 °C immediately after the milling was halted. In contrast, the synthesis of Z-Ala-Phg-Ile-OMe was carried out at the same temperature under traditional magnetic stirring conditions using the least quantity of DMF necessary to ensure adequate mixing (η = 20 μL/mg). Z-Ala-Phg-Ile-OMe was created under these experimental conditions with an 88% yield, 32% purity, and 9% LDL epimer (Table 1, entry 1). These findings unequivocally demonstrated that a C-terminal activated peptide fragment that was highly susceptible to epimerisation could be reduced through the use of liquid-assisted ball milling. Yebou et al. deduced from their findings that peptide fragment couplings can be achieved with high yields and extremely little epimerisation, if any, using a mixture of EDC-HCl, Oxyma, and modest amounts of a liquid additive (DMF or EtOAc) in a ball milling environment. Notably, excellent results were obtained with peptide segments that contained the C-terminal amino acids phenylglycine, cysteine, and valine, which are highly epimerisation-prone and/or severely hindered.

### 3.6. Microwave- and Flow Synthesis-Assisted

Automated flow peptide synthesis (AFPS) is a technique that greatly speeds up solid-phase chemical synthesis (SPSS) while maintaining its flexibility. In comparison to current human or automated approaches, AFPS can cut the amide bond-forming step for the fluorenylmethyloxycarbonyl (Fmoc) strategy in peptide synthesis and the full cycle for each amino acid addition to 40 s [93,94,95].

Cys and His amino acid epimerisation with high temperature flow activation was evaluated by Mijalis et al. in 2017. Cys and His can lose stereochemistry at the C position when activated, especially at high temperatures. A total of 16.7% of the undesirable D-Cys-containing product forms when Fmoc-L-Cys(Trt), HBTU, and DIEA are coupled for 1.5 min at 90 °C under microwave irradiation [96].

**Figure 43:**
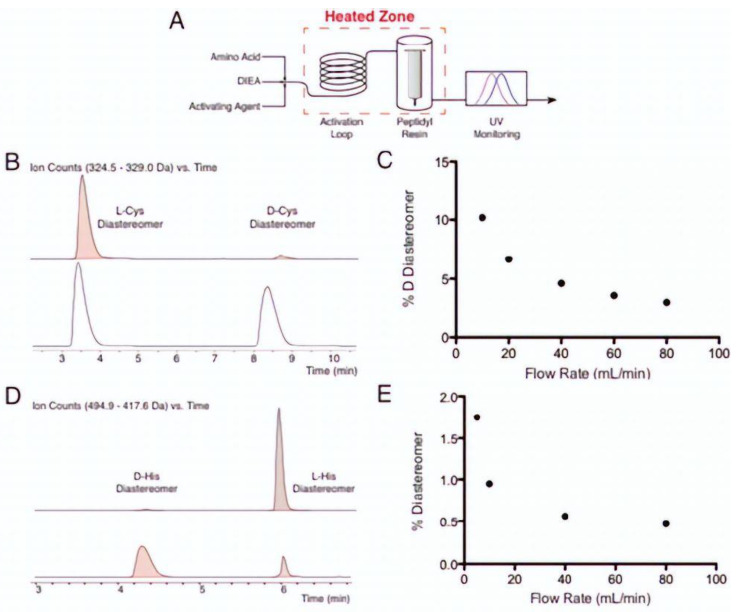
HPLC spectra in epimerisation study of microwave-assisted peptide synthesis. (**A**). Process flow diagram. Amino acid, activating agent, and DIEA are merged together using three HPLC pumps. (**B**). HPLC spectra of L-Cys and D-Cys residue. (**C**) Flow rate vs. %D dastereomer diagram of Cys residue. (**D**). HPLC spectra of L-His and D-His residue (**E**). Flow rate vs. %D dastereomer diagram of His residue.

As shown in Figure 43, however, they discovered that the epimerisation product of His and Cys amino acid can be suppressed with AFPS by increasing the flow rate, thereby reducing the residence time at high temperature for activating His and Cys monomers GCF, whose diastereomers are separable by RP-HPLC. They also discovered that, at 80 mL/min, 0.5% D-His and 3% D-Cys were incorporated. This level of diastereomer production is in accordance with optimised batch synthesis at room temperature.

In another study, Palasek et al. (2007) also evaluated the role of microwave-assisted peptide synthesis in limiting racemisation products in solid-phase peptide synthesis. A 20 mer peptide containing each of the natural 20 amino acids (VYWTSPFMKLIHEQCNRADG-NH_2_) was synthetised using a selectively placed C-terminal Asp-Gly segment. The reaction condition was carried out using a HBTU coupling agent with the present basic DIPEA in DMF. The reaction was compared with the conventional SPPS method [97].

From Table 21, it can be seen that, in conventional SPPS methods, almost all amino acids lead to epimerisation products; however, in small percentages, the racemisation in cysteine and aspartate still resulted in higher epimerisation (1.19% and 1.09%) than the other amino acid residue. The crude purity product of the conventional method was achieved at 68.4% in the microwave-assisted condition. The epimerisation product was not determined in most amino acid residue, and the epimerisation product was only found in cysteine (<1.14%) and histidine (0.67%), although it was still of low value. The crude purity product also increased with an obtained yield of 84.01%. From this result, it seems microwave energy can suppress the epimerisation product and enhance the yield of the desired product. 

## 4. Summary

Epimerisation is a side reaction that is widely found in peptide synthesis. Several factors induce the epimerisation, including the carboxylic group activation during the coupling step, the amino acid itself, the bases used, the steric factor and ring orientation present in the structure, the solvent employed, the temperature applied, and the cyclisation process applied. The selection of coupling agents and additives plays a very important role in the formation of epimerisation products in the activation of amino acids. Several amino acids, such as serine, cysteine, and methionine, have an EWG side chain that provides an induction effect upon H-alpha abstraction, causing epimerisation. The phenylglycine amino acid can cause a stabilising effect in the aryl side chain group to H-alpha and give the epimerisation a chance to occur. Several attempts can be made to suppress racemisation products, such as through selecting proper coupling reagents, selecting protecting groups, applying coupling strategies, providing assistance with physical mechanisms, and controlling reaction conditions.

## Figures and Tables

**Table 1:** Comparison of the epimerisation percentage of several amino acids by EDC and DCC.

	Ala (%)	Leu (%)	Phe (%)	Val (%)	Ile (%)
DCC/DCM	10	14	18	5	9
EDC/DCM	25	25	21	22	29
DCC-HOBt/DMF	0.8	6.0			
EDC-HOBt/DMF	2.0	9.0			

**Table 2:** Epimerisation percentage during activation using EDC, EDC-HCl, and DIC in the presence of HOAt.

Reagent	Percentage
EDC/HOAt	29.8
EDC-HCl/HOAt	24.1
DIC/HOAt	4.2

**Table 3:** Cyclic peptide mixtures, aspartic acid analogues, and their analysis by Marfey’s test.

No	Peptide	Mixture	Coupling Agent; Cyclisation Reagent	Mass Spectra FABMS; LCMS	*D-*Asp (%) Marfey’s Test (*L-*Asp)
1	C(Xxx-*D-*Leu-Val-*D-*Pro-Asp)	Xxx = Ala, Phe, Tyr, Trp	BOP/HOBt; BOP/HOBt	+; +	30.0
2	C(Xxx-*D-*Leu-Val-*D-*Pro-Asp)	Xxx = Phe, Tyr, Trp, Nal Yyy = Gly, Ala, Val, Leu	BOP/HOBt; BOP/HOBt	+; +	26.5
3	C(Xxx-*D-*Leu-Val-*D-*Pro-Asp)	Xxx = Ala, Phe, Tyr, Trp	Active ester; HATU/HOAt	+; +	10.7

**Table 4:** Cyclisation studies.

Entry	Coupling Reagent	Base	Solvent	pH conv	Diastereomeric Ratio (D: L)
1 2 3 4	PyBOP/HOAt PyBOP/HOAt PyBOP/HOAt DIPCDI/HOAt	- DIEA Collidine DIEA	DMF-DCM DMF-DCM DMF DMF-DCM	8 low 8 low 8 high 7	15:85 67:33 13:87

**Table 5:** Summary of yields and epimerisation rates for various peptide coupling conditions.

Coupling Condition (Equiv)		Fmoc-Ser(R)-OH, Where R =				
		Trt	Ac_3_GaINAca	Ac_4_GaIb1-3Ac_2_GaINAca	Ac_3_GlcNAca	Ac_3_GlcNAcb
AAs: 2.5; HATU/HOAt: 1.1/1.1; NMM 2.2; 0/3 h in DMF	Yield % (D/(D + L) %	75.1 0.8	69.1 5.1	83.9 4.3	100 7.7	69.0 8.1
AAs: 1.5; HATU/HOAt: 1.2/1.2; NMM 4.0; 0/8 h in DMF	Yield % (D/(D + L) %	81.0 2.2	74.6 11.4	96.7 21.2	89.9 15.0	92.0 20.0
AAs: 3.3; HATU/HOAt: 3.65/3.7; DIPEA 7.22; 0/12 h in DMF	Yield % (D/(D + L) %	98.9 0.2	99.4 0.8	83.0 65.6	100 72.5	1.95 2.0
AAs: 4.4; HATU/HOAt: 4.4/0; NMM: 8.8; 3/12 h in NMP	Yield % (D/(D + L) %	63.4 37.6	99.4 69.8	83.0 65.6	100 72.5	1.9 52.0
AAs: 5; HATU/HOAt: 6.25/6.25; NMM: 12.5; 0/6 h in NMP	Yield % (D/(D + L) %	98.4 2.9	99.4 5.2	98.8 6.5	99.5 8.1	95.2 9.7

**Table 6:** Epimerisation studies in Xaa*-Thi derivatives [36].

Reaction	Time (h)	Yield (%)
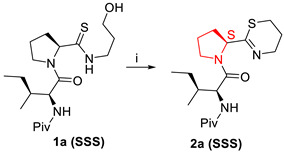	12 h	70
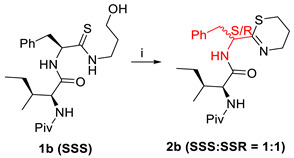	14 h	51
(i) MsCl (1.1 eq.), NEt_3_ (2 eq.), THF (0.06 M), 0 °C–rt.		

**Table 7:** Epimerisation ratio during activation in difference bases and solvents.

Entry	Base	Solvent	pH Conv.	Diastereomeric Ratio (D:L)
1	DIEA	DMF-DCM	8 low	15:85
2	Collidine	DMF-DCM	8 low	67:33
3	DIEA	DMF	8 high	13:87

**Table 8:** Epimerisation ratio during activation in difference base.

Amine		Xxx (%)		
Base	Equiv	Phe	Leu	Val
DIPEA	1	2.4		
DIPEA	3	11.4		
DIPEA	2	3.7	2.5	1.7
Bu_3_N	2	4.1		
TEA	2	5.3		2.4
NMP	2	7.8		
NMM	2	9.6	8.7	2.7
NMM	1	4.4		
NMM	3	15.0		

**Table 9:** Level of epimerisation in the tripeptide deprotection step.

Incubation Solution	Level of Epimerisation (*D-*Peptide/*L-*Peptide) × 100
None	1.39%
20% piperidine	1.56%
5% piperazine + 2% DBU	1.91%
5% piperazine + 1% DBU + 1% FA	1.41%

**Table 10:** Effect of addition of DBU and/or formic acid on aspartimide and piperidide/piperazide formation in resin bound VKDGYI.

Incubation Solution	Relative Yield %		
	Target Peptide	*D/L-*Aspartimide	Piperidides or Piperazides
No treatment	97.6	2.4	n.d.
20% piperidine	55.6	24.4	20.0
20% piperidine + 1% FA	77.5	17.2	5.3
5% piperazine + 1% DBU	9.2	13.7	77.1
5% piperazine + 1% FA	82.8	7.2	n.d.
5% piperazine + 1% DBU + 1% FA	86.0	14.0	n.d.

**Table 11:** Rate of epimerisation based on ring size.

No	Peptide Name	Sequence	Ring	% D to L
1	[1–8-NαC],[1-MetO_2_]-linusorb B1q	MetO_2_-Leu-Val-Phe-Pro-Leu-Phe-Ile	8	61.16%
2	[1–9-NαC],[1-MetO_2_]-linusorb B2	MetO_2_-Leu-Ile-Pro-Pro-Phe-Phe-Val-Ile	9	20.48%
3	[1–8-NαC],[1-(Rs,Ss)-MetO]-linusorb B1	[(R,S)-MetO]-Leu-Val-Phe-Pro-Leu-Phe-Ile	8	55.28%
4	[1–9-NαC],[1-(Rs,Ss)-MetO]-linusorb B2	[(R,S)-MetO]-Leu-Ile-ProPro-Phe-Phe-Val-Ile	9	29.43%

**Table 12:** Data summary of Diastereoselective Excesses (deL) and kinetic constants for epimerisation (kr) of Phg residue at the N-terminal.

Variable	Phg-Ala-OH	Phg-Val-OH	Phg-Ala-Ser-NH-Me	Phg-Val-Ser-NH-Me	Phg-Ala-NH
de_L_ (∞) (%)	−13	−42	−1	−16	−15
kr_DL_ (10^−5^ s^−1^)	0.54	0.19	0.50	-	3.9
kr_LL_ (10^−5^ s^−1^)	0.70	0.46	0.51	-	5.3

**Table 13:** % D to L conversion of LOs.

LO	Area under	LO	Area under	% D to L Conversion
	Curve		Curve	
2	574.834	8	31.1244	5.14
3	2314.17	9	964.877	29.43
4	1251.1	10	322.223	20.48
5	767.894	11	227.048	22.82
6	411.138	12	508.19	55.28
7	319.425	13	502.883	61.16
**LOCode**	**Amino acid** **sequence (NaC-)**		**Molecular formula**	**MW (Da)**
1–9-NαC]-linusorb B3 (**1**)	Ile-Leu-Val-Pro-Pro-Phe-Phe-Leu-Ile		C_57_H_85_N_9_O_9_	1040.34
[1–9-NαC]-linusorb B2 (**2**)	Met-Leu-Ile-Pro-Pro-Phe-Phe-Val-Ile		C_56_H_83_N_9_O_9_S	1058.38
[1–9-NαC],[1-(Rs,Ss)-MetO]-linusorb B2 (**3**)	[(Rs,Ss)-MetO]-Leu-Ile-Pro-Pro-Phe-Phe-Val-Ile		C_56_H_83_N_9_O_10_S	1074.38
[1–9-NαC],[1-MetO_2_]-linusorb B2 (**4**)	MetO2-Leu-Ile-Pro-Pro-Phe-Phe-Val-Ile		C_56_H_83_N_9_O_11_S	1090.38
[1–8-NαC]-linusorb B1 (**5**)	Met-Leu-Val-Phe-Pro-Leu-Phe-Ile		C_51_H_76_N_8_O_8_S	961.26
[1–8-NαC],[1-(Rs,Ss)-MetO]-linusorb B1 (**6**)	[(Rs,Ss)-MetO]-Leu-Val-Phe-Pro-Leu-Phe-Ile		C_51_H_76_N_8_O_9_S	977.26
[1–8-NαC],[1-MetO_2_]-linusorb B1 (**7**)	MetO2-Leu-Val-Phe-Pro-Leu-Phe-Ile		C_51_H_76_N_8_O_10_S	993.26
[1–9-NαC], DMet-linusorb B2 (**8**)	DMet-Leu-Ile-Pro-Pro-Phe-Phe-Val-Ile		C_56_H_83_N_9_O_9_S	1058.38
[1–9-NαC],[1-(Rs,Ss)-DMetO]-linusorb B2 (**9**)	[(Rs,Ss)-DMetO]-Leu-Ile-Pro-Pro-Phe-Phe-Val-Ile		C_56_H_83_N_9_O_10_S	1074.38
[1–9-NαC],[1-DMetO_2_]-linusorb B2 (**10**)	DMetO_2_-Leu-Ile-Pro-Pro-Phe-Phe-Val-Ile		C_56_H_83_N_9_O_11_S	1090.38
[1–8-NαC], DMet-linusorb B1 **(11)**	DMet-Leu-Val-Phe-Pro-Leu-Phe-Ile		C_51_H_76_N_8_O_8_S	961.26
[1–8-NαC],[1-(Rs,Ss)-DMetO]-linusorb B1 (**12**)	[(Rs,Ss)-DMetO]-Leu-Val-Phe-Pro-Leu-Phe-Ile		C_51_H_76_N_8_O_9_S	977.26
[1–8-NαC],[1-DMetO_2_]-linusorb B1 (**13**)	DMetO_2_-Leu-Val-Phe-Pro-Leu-Phe-Ile		C_51_H_76_N_8_O_10_S	993.26

**Table 14:** Racemisation studies on the solid-phase assembling of H-GlyAA-Phe-NH_2_ (where AA = Ser, Cys, Cys(Acm) or His).

Entry	Coupling Model	Coupling Reagent	DL/LL (%)
1	H-Gly-Ser-Phe-NH_2_	DIC/HOBt	3.3
2		DIC/HOAt	0.4
3		DIC/OxymaPure	0.4
4		DIC/Oxyma-B	0.4
5	H-Gly-Cys-Phe-NH_2_	DIC/HOBt	0.5
6		DIC/HOAt	0.4
7		DIC/OxymaPure	0.3
8		DIC/Oxyma-B	0.3
9	H-Gly-Cys(Acm)-Phe-NH_2_	DIC/HOBt	0.4
10		DIC/HOAt	0.3
11		DIC/OxymaPure	0.3
12		DIC/Oxyma-B	0.3
13	H-Gly-His-Phe-NH_2_	DIC/HOBt	1.1
14		DIC/HOAt	1.9
15		DIC/OxymaPure	3.0
16		DIC/Oxyma-B	1.0

**Table 15:** Coupling agent effect in minimising % epimerisation.

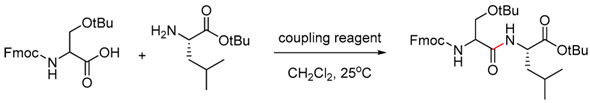					
Entry	Coupling Reagent	Additive	Time	Yield	Dr
**1**	HBTU	DIEA	10 min	90%	82:18
**2**	HATU	DIEA	10 min	70%	87:13
**3**	PyBop	DIEA	10 min	91%	88:12
**4**	DCC	--	10 min	98%	91:9
**5**	DEPBT	DIEA	20 min	61%	99:1
**6**	MYMsA	--	22 h	99%	100:0
**7**	MYTsA	--	22 h	94%	100:0

**Table 16:** Epimerisation study in DBAA mediated coupling agent SPPS.

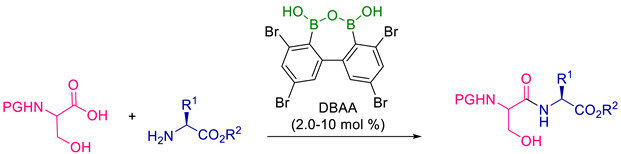					
Entry	PG	R1	R2	Yield	De (%)
1	Boc	Gly	Bn	88	99
2	Fmoc	Gly	Et	81	99
3	Cbz	Ala	^t^Bu	79	99
4	Boc	Leu	Me	99	99
5	Cbz	Leu	Me	98	99
6	Boc	Ile	Me	96	98
7	Fmoc	Ile	Me	74	98
8	Boc	Val	Me	99	99
9	Cbz	Val	Me	88	99
10	Boc	^t^Bu	Me	97	98
11	Boc	Me_2_	Bn	21	93
12	Boc	(N-Me) Gly	Bn	47	96
13	Cbz	Tyr (O-^t^Bu)	Me	79	99
14	Boc	Tyr (O-^t^Bu)	Me	85	99
15	Fmoc	Tyr (O-^t^Bu)	Me	79	99
16	Fmoc	Tyr	Me	68	98
17	Boc	Asp (O-^t^Bu	Me	97	99
18	Fmoc	Trp	Me	79	96
19	Boc	Lys (Boc)	Me	99	96
20	Boc	Arg (Pbf)	Me	72	99
21	Boc	His (Trt)	Me	64	98
22	Boc	Met (Me)	Me	81	99
23	Boc	Cys (Bn)	Me	75	99

**Table 17:** Epimerisation study in Copper(II)-mediated Chan–Lam-type coupling.

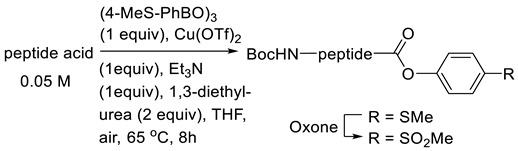					
Entry	Peptide Acid	R = SMe		R = SO_2_Me	
		Yield (%)	de (%)	Yield (%)	de (%)
1	Boc-Trp-Phe-OH	57	n.d. *	-	-
2	Boc-Phe-Phe-OH	77	99.9	97	99.9
3	Boc-Phe-*D-*Phe-OH	75	99.9	quant	99.9
4	Boc-Phe-Phg-OH	70	99.9	quant	99.9
5	Boc-Phe-*D-*Phg-OH	73	99.9	95	99.9
6	Boc-Phe-Val-OH	68	n.d.	quant	n.d.
7	Boc-Trp(Boc)-Val-OH	70	n.d.	quant	n.d.
8	Boc-Phe-Ala-OH	83	n.d.	quant	n.d.
9	Boc-Asp-(^t^Bu)-Phe-Phe-OH	55	n.d.	quant	n.d.

* n.d: not detected.

**Table 18:** Epimerisation study in an organic salt additive coupling condition.

Inorganic Additive (Equiv.)	Z-I-Ala-*D*-Phe-Gly-OBzl (%)			Z-I-Ala-*D*-Phe-Gly-OH (%)		
	0.5	1	2	0.5	1	2
None		3.0		2.7		
LiCl	2.7	1.2	5.4	2.0	1.0	4.8
NaI	1.7	<1.0	2.4	2.2	1.5	4.8
RbClO_4_	1.5	<1.0	1.8	2.7	<1.0	1.6
CsF	3.3	2.5	5.2	3.0	2.7	5
Mg(ClO_4_)_2_	4.0	3.5	5.4	4.7	4	6.6
CaCl_2_	3.6	2.8	4.2	4.3	3.0	5.7
BaI_2_	3.7	3.0	5.2	4.3	4.0	6
BF_3_gt;Et_2_O	1.6	<1.0	1.4	2.0	<1.0	1.7
ZnCl_2_	2.4	<1.0	3.1	2.0	<1.0	3.0
SnCl_4_	1.8	<1.0	2.1	1.5	<1.0	2.2
AlCl_3_	1.8	<1.0	1.5	1.4	<1.0	1.2
CdI_2_	2.3	2.0	2.7	3.0	1.6	3.4
CuCl_2_	<0.1	<0.1	<0.1	<0.1	<0.1	<0.1

**Table 19:** Screening of silylating reagents in the peptide bond-forming reaction.

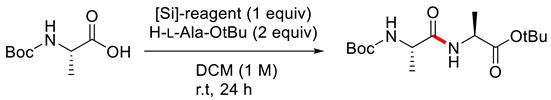			
Entry	[Si]-Reagent	Yield of 1 (%)	dr
1	ClSi(OEt)_3_	43	97:3
2	Si(OCH_2_CF_3_)_4_	53	>99:1
3	Si(OCH(CF_3_)_2_)_4_	66	>99:1
4	HSi(OCH_2_CF_3_)_3_	63	>99:1
5	HSi(OCH_2_CCl_3_)_3_	53	>99:1
6	HSi(OCH(CF_3_)_2_)_3_	78	>99:1
7	HSi(OCH_2_CF_2_CHF_2_)_3_	53	>99:1
8	HSi(OCH(CF_3_)_2_)_3_	95	>99:1

**Table 20:** Epimerisation study of ball-milling-assisted peptide containing PhG peptide.

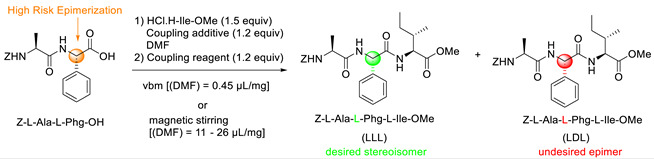						
Entry	Reagent	Temperature (°C)	Time, t (min)	Yield (%)	Purity (%)	LDL (%)
1	EDC.HCl/Oxyma	33 (33)	10 (30)	93(88)	>99 (32)	<1 (9)
2	EDC.HCl/HOBt.H_2_O	34 (34)	10 (30)	90 (90)	70 (48)	25 (35)
3	EDC.HCl/HOAt	34 (34)	10 (30)	88 (90)	95 (59)	<1 (26)
4	DIC/HOAt	30 (31)	10 (40)	n.d (n.d)	67 (39)	17 (33)
5	DIC/Oxyma	31 (31)	10 (40)	n.d (n.d)	46 (<10)	<1 (n.d)
6	HATU/Et_3_N	34 (34)	10 (60)	85 (88)	88 (58)	1 (<1)
7	HBTU/Et_3_N	33 (33)	10 (20)	86 (82)	71 (55)	2 (9)
8	EDC.HCl/Oxyma	n.d	30	96	>99	<1

**Table 21:** Crude product purity measured by LC-MS and racemisation of amino acids measured by GC-MS after hydrolysis of VYWTSPFMKLIHEQCNRADG-NH_2_ with 6 N DCl/D_2_O.

Amino Acid	Synthesis Condition	
	Conventional HBTU/DIPEA in DMF	Microwave HBTU/HOBT in DMF (80 °C)
*D-*Asp	1.19	No detection
*D-*Ala	0.21	No detection
*D-*Arg	0.18	No detection
*D*-Cys	1.09	<1.14
*D*-Glu	1.46	No detection
*D*-His	0.65	0.67
*D*-Ile	<0.1	No detection
*L*-allo Ile	<0.1	No detection
*D*-allo Ile	<0.1	No detection
*D*-Leu	0.17	No detection
*D*-Lys	0.1	No detection
*D*-Met	0.48	No detection
*D*-Phe	0.28	No detection
*D*-Pro	<0.1	No detection
*D*-Ser	0.46	No detection
*D*-Thr	<0.1	No detection
*L*-allo Thr	<0.1	No detection
*D*-allo Thr	<0.1	No detection
*D*-Trp	0.19	No detection
*D*-Tyr	0.42	No detection
*D*-Val	<0.1	No detection
Crude product purity	68.4%	84.0%
Synthesis time (h)	23.3	10.1

## Data Availability

Not applicable.
